# Theory of photosynthetic membrane influence on B800-B850 energy transfer in the LH2 complex

**DOI:** 10.1016/j.bpj.2025.10.032

**Published:** 2025-10-30

**Authors:** Chawntell Kulkarni, Hallmann Óskar Gestsson, Lorenzo Cupellini, Benedetta Mennucci, Alexandra Olaya-Castro

## Main text

(Biophysical Journal *124*, 722–739; March 4, 2025)

After publication, the authors became aware of a sign mistake in the numerical implementation of the modified Redfield rates determining the exciton lifetimes used in the GFT calculations. Consequently, the width of the GFT distributions presented in Figures 6 *a* and 7 change, leading to a closer qualitative agreement between GFT and the HEOM-fitted rates (Fig. 6 *b*, which remains unchanged). Some quantitative values in Tables 4 and 5 are also altered, but the qualitative differences still hold. These changes do not affect the main conclusions of the paper, namely that electronic quantum properties are the main factor underlying the faster rate and broader distributions in the membrane. The authors apologize for this error.

The main corrections and their impact are as follows:

• **Equation:** In the Supporting Material, Equation 2 contained a sign error in the exponential: instead of “−*iω*_*αβ*_*t*,” it should read “+*iω*_*αβ*_*t.*” The equation has been corrected in the Supporting Material file.
• **Main text:** Figures 6 and 7 (and their corresponding legends) and Tables 4 and 5 have been corrected online. Average GFT rates are now in closer agreement with the HEOM-fitted rates reported in the original article, and the GFT distributions are somewhat narrower. In particular, GFT now predicts faster transfer in the DOPC membrane than in detergent, consistent with HEOM and experiment. The corrected rates indicate that the broader distributions in the POPC membrane arise primarily from excitonic coupling rather than differences in spectral overlap. Importantly, these changes do not alter the main conclusions: electronic properties remain the key factor underlying the faster transfer rates and broader distributions in the membrane.
  Tables 4 and 5 show corrected values for *P*_*α*_*k*_*αβ*_, *k*_*αβ*_, and *O*_*αβ*_. These quantitative differences arise from the corrected exciton lifetimes, but the qualitative differences remain unchanged. Exciton coupling and coherence norms are also unchanged.
• **Supporting Material:** Figures S2 and S3 and Table S1 have been corrected in the Supporting Material file. These updates rescale distributions and averages, but all qualitative trends and control checks (e.g., comparisons using identical static disorder or identical spectral densities) remain unchanged. Comparisons of average rates across alternative detergent models still support our chosen model as the best representation of experimental observations.

In summary, the correction leads to a better agreement between GFT and HEOM while still showing the slight differences between the predictions from the two frameworks. The main conclusions of the work are not altered. The new figures indicate that GFT is good enough for qualitative comparisons of average LH2 inter-band rates in different environments but still falls short for accurate predictions of the (left) skewness of the rate distributions. This means that GFT fails to properly capture the contributions of electronic configurations that lead to slow transfer rates.

Benchmarking code reproducing Yang and Fleming’s results (Supporting Material (1)) is provided in our GitHub repository (Supporting Material (2)). The folder “benchmarking/” contains the script “modified_redfield_benchmark.py,” which runs the current implementation of the modified Redfield rates and reproduces Fig. 2 from (Supporting Material (1)), verifying the corrected expression.

**Figure:**
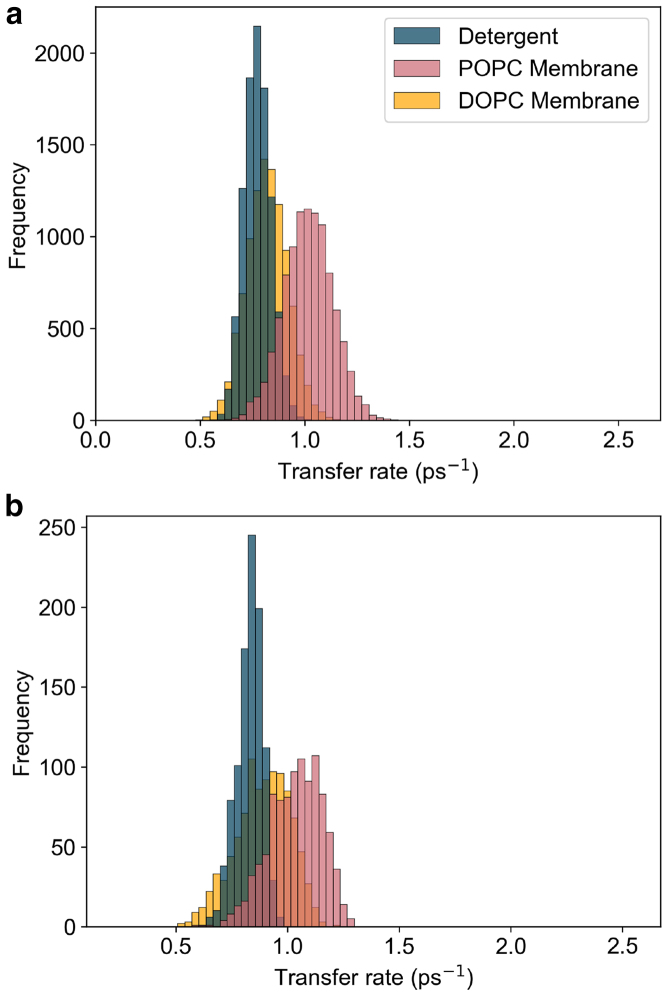
Figure 6. B800 to B850 energy transfer rate distribution (corrected)

**Figure:**
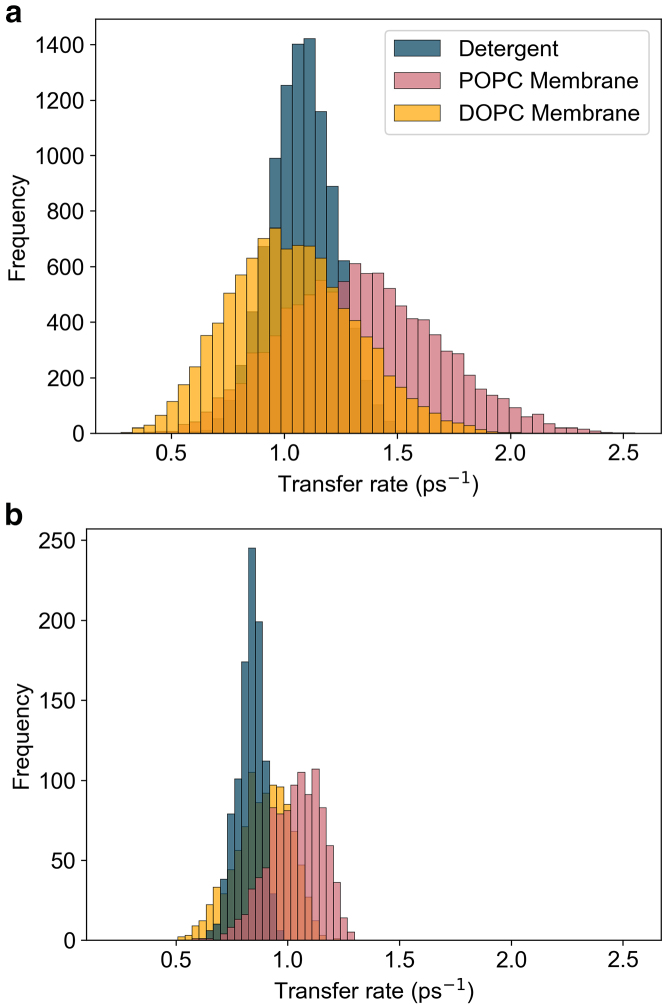
Figure 6. B800 to B850 energy transfer rate distribution (original)

**Figure:**
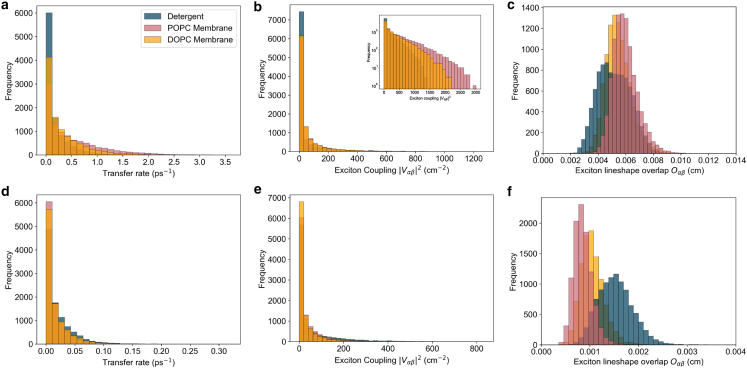
Figure 7. Distribution of key exciton properties (corrected)

**Figure:**
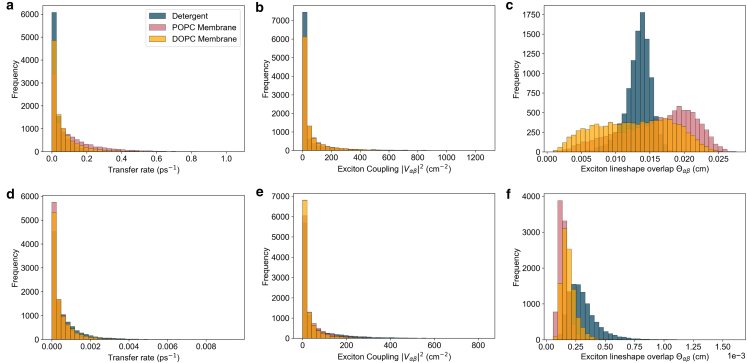
Figure 7. Distribution of key exciton properties (original)

**Figure:**
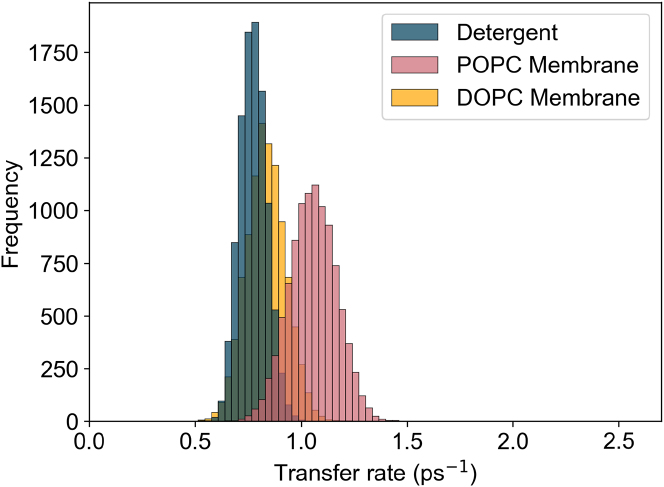
Figure S2. Distributions of 10,000 realisations of the B800 to B850 transfer rate using GFT for detergent, DOPC, and POPC, using identical static disorder parameters (corrected)

**Figure:**
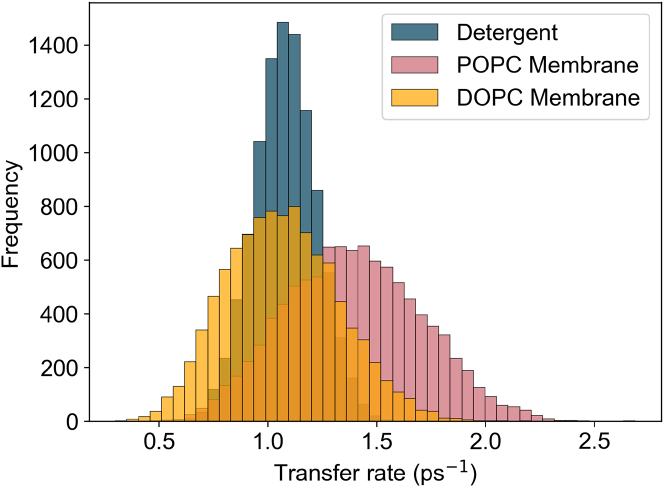
Figure S2. Distributions of 10,000 realisations of the B800 to B850 transfer rate using GFT for detergent, DOPC, and POPC, using identical static disorder parameters (original)

**Figure:**
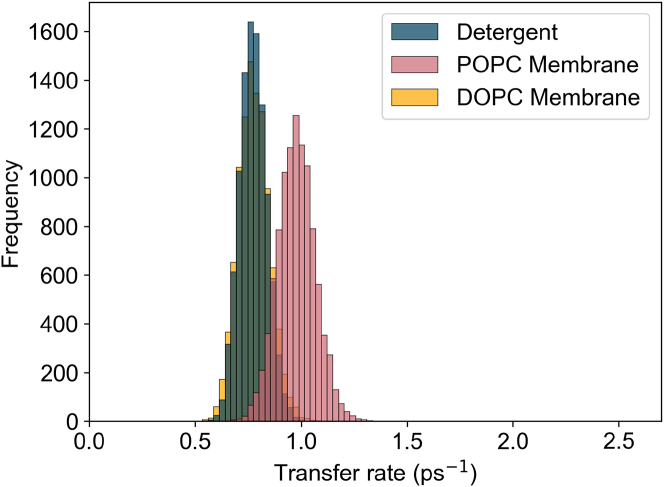
Figure S3. Distributions of 10,000 realisations of the B800 to B850 transfer rate using GFT for detergent, DOPC, and POPC using an identical spectral density (corrected)

**Figure:**
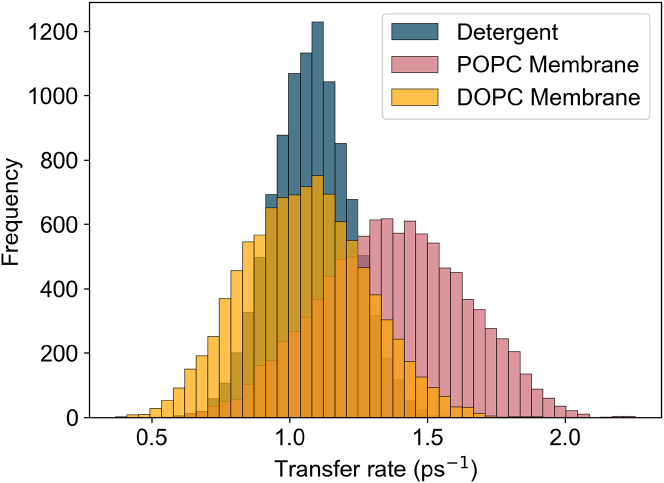
Figure S3. Distributions of 10,000 realisations of the B800 to B850 transfer rate using GFT for detergent, DOPC, and POPC using an identical spectral density (original)

