# Supplementary material for: Theory of photosynthetic membrane influence on B800-B850 energy transfer in the LH2 complex
Source: Biophys J. 2025 Jan 22;124(5):722–39. doi: 10.1016/j.bpj.2025.01.011 (PMC11897548; doi:10.1016/j.bpj.2025.01.011)
Supplement: Document S1. Figures S1–S5 and Tables S1–S3 [file mmc1.pdf]

Biophysical Journal, Volume *124*

Supplemental information

Theory of photosynthetic membrane influence on B800-B850 energy transfer in the LH2 complex

Chawntell Kulkarni, Hallmann Óskar Gestsson, Lorenzo Cupellini, Benedetta Mennucci, and Alexandra Olaya-Castro

Supporting material

Theory of photosynthetic membrane influence on B800-B850 energy transfer in the LH2 complex

Chawntell Kulkarni[1], Hallmann Óskar Gestsson[2], Lorenzo Cupellini[3], Benedetta Mennucci[4], and Alexandra Olaya-Castro[5]

[1, 2, 5]Department of Physics and Astronomy, University College London, London WC1E 6BT, United Kingdom

[3, 4]Dipartimento di Chimica e Chimica Industriale, Università di Pisa, Via G. Moruzzi 13, 56124 Pisa, Italy

Modified Redfield theory

The energy transfer rates computed with generalised Förster theory (GFT) use lineshape functions that are derived perturbatively as introduced in the generalised Förster theory section. The exciton lifetime τ_α that enters the lineshape functions is given by

$$\tau_\alpha = \left(\frac{1}{2} \sum_{\alpha \neq \beta} k_{\alpha\beta}^{\text{MR}} \right)^{-1}, \tag{1}$$

where $k_{\alpha\beta}^{\text{MR}}$ is the energy transfer rate from a donor exciton α to all possible acceptor excitons β, given that they are localised on the same ring as the donor exciton. Since the interchromophore electronic couplings within each ring are strong, modified Redfield theory is used to obtain the intra-ring exciton transfer rates. By assuming that the electronic states of each ring are weakly coupled to their environment, H_{SB} is treated as a perturbation on the dynamics within each ring.

The modified Redfield energy transfer rate between two excitons in the same ring is given by [1]

$$\begin{aligned} k_{\alpha\beta}^{\text{MR}} = 2\text{Re} \int_0^\infty dt \; & e^{-i\omega_{\alpha\beta}t} e^{-i(\lambda_{\alpha\alpha,\alpha\alpha}+\lambda_{\beta\beta,\beta\beta})t} e^{-g_\alpha(t)-g_\beta(t)} e^{2g_{\beta\beta,\alpha\alpha}+2i\lambda_{\beta\beta,\alpha\alpha}} \\ & \times \left[\ddot{g}_{\beta\alpha,\beta\alpha}(t) - \left(\dot{g}_{\beta\alpha,\beta\beta}(t) - \dot{g}_{\beta\alpha,\alpha\alpha}(t) + 2i\lambda_{\beta\alpha,\beta\beta}\right)^2 \right], \end{aligned} \tag{2}$$

where the terms have been defined in the main text (generalised Förster theory section).

Propagation of the dipole operator

Numerical computation of the absorption and fluorescence expressions given in the Linear spectra section using HEOM theory is achieved by rewriting the auto-correlation as

$$\langle \hat{\mu}_p(t)\hat{\mu}_p(t)\rangle_\rho = \mathrm{Tr}(\hat{\mu}_p e^{\mathcal{L}t}[\hat{\mu}_p\hat{\rho}]), \tag{3}$$

where $\mathcal{L}$ is the HEOM generator of dynamics and $\hat{\rho}$ is the reduced system density matrix. The half-sided Fourier transform is then formally calculated to give

$$\int_0^\infty \mathrm{d}t\hat{\mu}_p e^{\mathcal{L}t}[\hat{\mu}_p\hat{\rho}]e^{i\omega t} = -\hat{\mu}_p\frac{1}{\mathcal{L}+i\omega}[\hat{\mu}_p\hat{\rho}]. \tag{4}$$

We numerically determine $\hat{x}_{p,\omega} = \frac{1}{\mathcal{L}+i\omega}[\hat{\mu}_p\hat{\rho}]$ by solving the linear system $(\mathcal{L}+i\omega)[\hat{x}_{p,\omega}] = \hat{\mu}_p\hat{\rho}$ using the BiCGSTAB Krylov subspace method [2]. This method of numerically computing spectra is more efficient than numerically Fourier transforming the dynamics as a result of the sparsity of the matrix representation for $\mathcal{L}$.

Finding the thermal state

The fluorescence spectra in the Linear spectra section is computed by performing a trace with respect to the thermal state $\hat{\rho}_{th}$, which satisfies the property $\mathcal{L}\hat{\rho}_{th} = 0$. In order to determine the thermal state we solve this linear system using the BiCGSTAB method [2] which is supplied with an initial guess given by the Boltzmann state $e^{-\beta\hat{H}}/\mathrm{Tr}(e^{-\beta\hat{H}})$. Doing so guarantees that the solver will not yield the trivial zero matrix solution, which of course does not represent a physical state.

Donor acceptor exciton energy gap

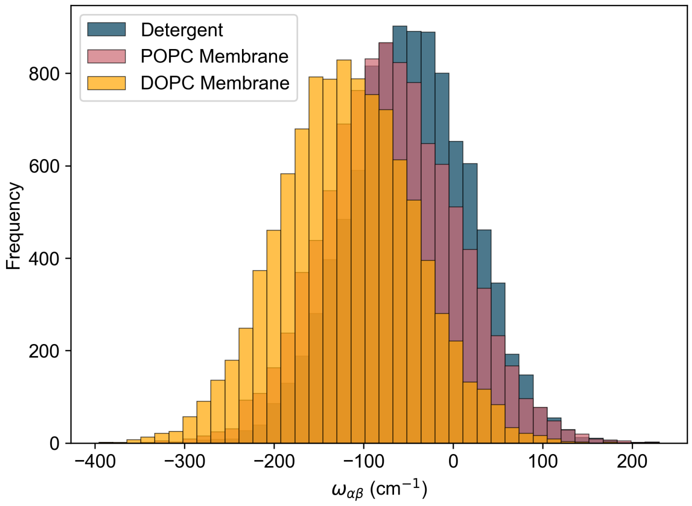

Figure S1: Distribution of 10,000 realisations of the energy gap between the B800 and B850 exciton forming the dominant exciton energy transfer pathway in detergent isolated LH2, LH2 embedded in a DOPC membrane and in a POPC membrane. Despite different levels of static disorder in membrane and detergent environments, the distribution of the energy gap of the dominant pathway in B800 to B850 transfer remains similar in each environment.

B800 to B850 transfer rate distributions

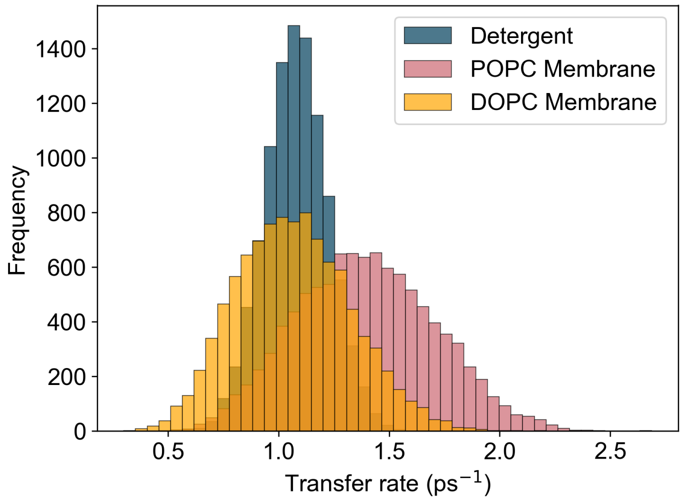

Figure S2: Distribution of 10,000 realisations of the B800 to B850 energy transfer rate calculated using GFT for detergent isolated LH2, and two different lipid compositions of membrane embedded LH2, DOPC and POPC. The same static disorder parameters were used to calculate rates in all three environments. Average transfer rates are 1.08 ps^{-1}, 1.07 ps^{-1} and 1.40 ps^{-1} respectively.

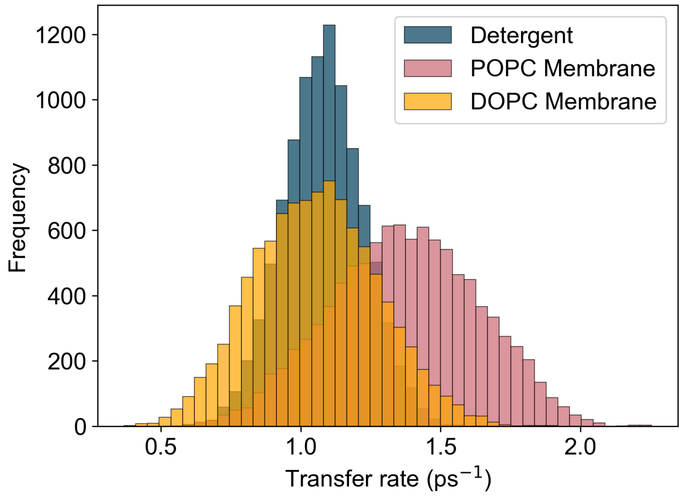

Figure S3: (a) Distribution of 10,000 realisations of the B800 to B850 energy transfer rate calculated using GFT for detergent isolated LH2, and two different lipid compositions of membrane embedded LH2, DOPC and POPC. The same spectral density was used for all three environments. Average transfer rates are 1.08 ps^{-1}, 1.05 ps^{-1} and 1.37 ps^{-1} respectively.

Linear spectra for alternative Hamiltonian parameters

To select Hamiltonian parameters that describe LH2 in detergent, we conducted an extensive review of the different parameter sets reported in the literature. For membrane LH2, to our knowledge, no reliable parameter sets other than the ones reported by some of us [3, 4] are available. Parameters for detergent-isolated LH2 computed using the same quantum chemical methods as those used for membrane-embedded LH2 are currently not available, and their computation requires additional developments outside the scope of this study. Existing parameter sets are either derived from first principles or from experimental spectroscopic measurements [5]. To assess which parameter set is suitable to describe detergent-solubilised LH2, we compare the linear spectra predicted by each set to what is measured in experimental. Specifically, we compare the predicted redshift of the B850 absorption peak in membrane relative to the peak position in detergent, as this is the key observation in experimental work comparing LH2 solubilised in detergent to LH2 in a lipid environment [6, 7, 8].

We have selected parameter sets for LH2 from the literature that have each been derived using different standard methodologies and compute linear absorption and fluorescence spectra using the hierarchical equations of motion (HEOM). We compare parameters derived using point dipole approximation (PDA) [9], point monopole approximation (PMA) [10] and transition density cubes method (TDCM)[11] to the parameters used in our study which have been derived from experimental spectra of the LH2 [12] The PDA and TDCM derived parameters do not have site energy values hence we use the site energies from the detergent model used in the paper. The most important aspect are the electronic couplings which we have shown underlie the observed changes in energy transfer rates and spectra from detergent to membrane.

Figure S4(a) shows the experimental spectra extracted from Figure 1(b) in the study by Ogren *et al.* [6] and Figures S4(b-d) show the spectra computed using each of the detergent parameter sets (shown in green) along with the spectra computed with the detergent and membrane parameters used in our study (shown in blue and red, respectively).

All three parameter sets predict the redshift of the B850 absorption peak in membrane compared to detergent despite each being derived using different methodologies. However, the predicted size of the redshift varies from one model to another which may result in changes in the B800 to B850 transfer rates that each model predict. We find that the size of the redshift predicted by the detergent model that we use in our study best compares with the experimentally observed shift.

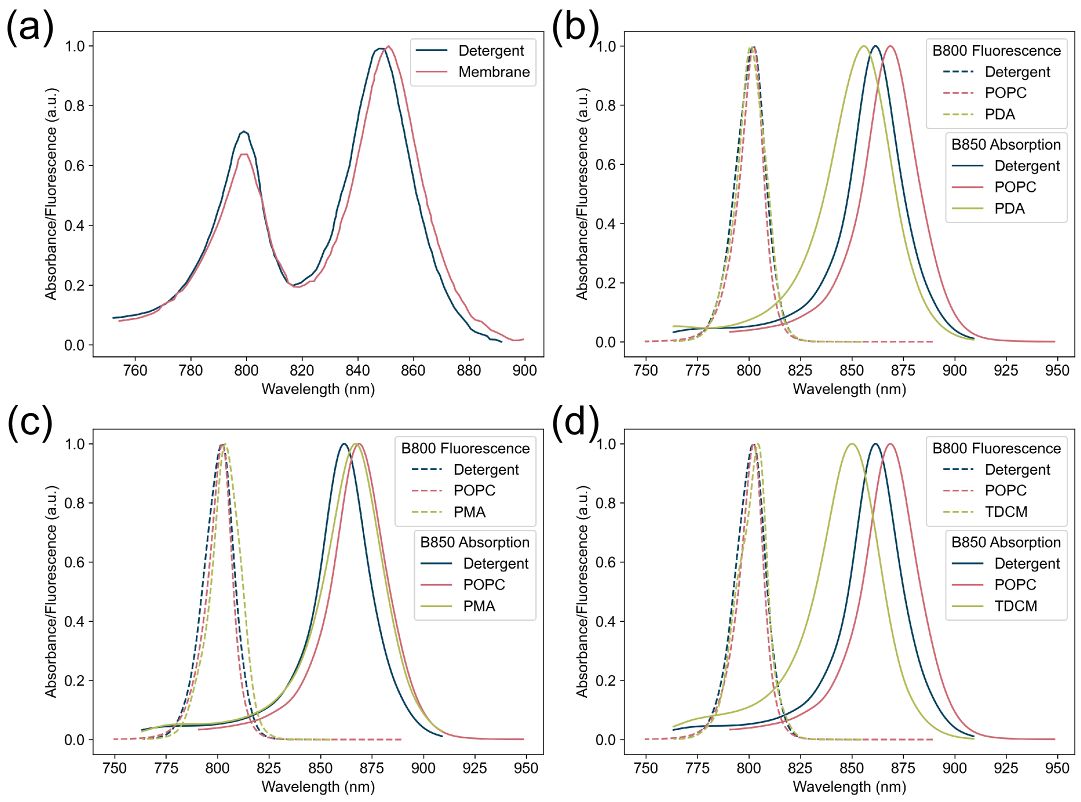

Figure S4: (a) Experimental absorption spectra of the LH2 from *Rhodobacter sphaeroides* in detergent and in membrane extracted from Figure 1(b) in Ogren *et al.* [6]. (b-d) Absorption spectra of the B850 ring and fluorescence spectra of the B800 ring for the Detergent and POPC membrane models used in the paper calculated using HEOM with truncation 3 for absorption and truncation 4 for fluorescence. The spectra are averaged over 1000 realisations of static disorder. For comparison a second detergent model is given in each plot where the electronic parameters have been computed using (b) point dipole approximation [9], (c) point monopole approximation [10] (d) and transition density cubes method [11].

B800 to B850 energy transfer rate for alternative Hamiltonian parameters

In the previous section, we showed how different Hamiltonian parameters predict varying degrees of redshift of the B850 peak from detergent to membrane, the redshift being the key observation in experimental spectroscopic measurements. The varying position of the B850 band predicted by each detergent parameter set, mean changes in the overlap of the B800 fluorescence and B850 absorption, which may result in differences in the B800 to B850 transfer rate predicted by each model.

Experimental pump-probe measurements find a 30% increase in B800 to B850 transfer rate from LH2 in detergent to LH2 in a lipid environment [6]. Using GFT to compute the B800 to B850 transfer rate averaged over 10,000 realisations, we compare the change in rate from detergent to membrane predicted by each of the detergent parameter sets, to what is measured in experiment.

Table S1 lists the B800 to B850 transfer rate predicted by each of the detergent parameter sets and the percentage change in transfer rate from detergent to membrane POPC. The alternative parameter sets show significant qualitative differences compared to experimental findings. The PMA and PDA parameters predict that the average transfer rate nearly doubles from detergent to membrane, while the TDCM parameters predict that energy transfer is actually slower in membrane. In contrast, the detergent parameters chosen for our study predict a 24% increase in the transfer rate, in qualitative agreement with experimental observations. Thus, the chosen set of parameters for detergent qualitatively capture the fundamental spectral and dynamical changes in the LH2 from detergent to lipid membrane environments observed in experiment, making the selected model an ideal basis to advance our understanding of the microscopic changes occurring within the complex in it's native physiological environment.

Parameter set	Membrane POPC	Detergent	PMA	PDA	TDCM
Transfer rate (ps^{-1})	1.34	1.08	0.69	0.67	1.52
% increase in rate in membrane	n/a	24%	94%	100%	-11%

Table S1: Average B800 to B850 transfer rates computed using GFT with 10,000 realisations of disorder for various Hamiltonian parameters. The parameter sets are, as listed from left to right, the membrane POPC and detergent parameters used in our study, alternative detergent parameters computed using point monopole approximation, point dipole approximation and transition density cubes method. The last row gives the percentage change in transfer rate from each of the alternative detergent models to membrane POPC.

Standard deviation of exciton energies

Exciton	Membrane	Detergent
k = 9	111	93
k = +8	87	73
k = -8	80	67
k = +7	76	64
k = -7	75	62
k = +6	74	62
k = -6	73	61
k = +5	73	62
k = -5	72	64
k = +4	71	60
k = -4	72	59
k = +3	71	59
k = -3	74	60
k = +2	72	58
k = -2	75	60
k = +1	74	59
k = -1	79	63
k = 0	91	65

Table S2: Standard deviations of the B850 exciton energy levels in cm^{-1} computed using 10,000 realisations of disorder listed from highest to lowest energy.

Exciton	Membrane	Detergent
D = 8	19	28
D = 7	16	22
D = 6	15	20
D = 5	15	19
D = 4	15	19
D = 3	15	19
D = 2	15	20
D = 1	16	21
D = 0	19	26

Table S3: Standard deviations of the B800 exciton energy levels in cm^{-1} computed using 10,000 realisations of disorder listed from highest to lowest energy.

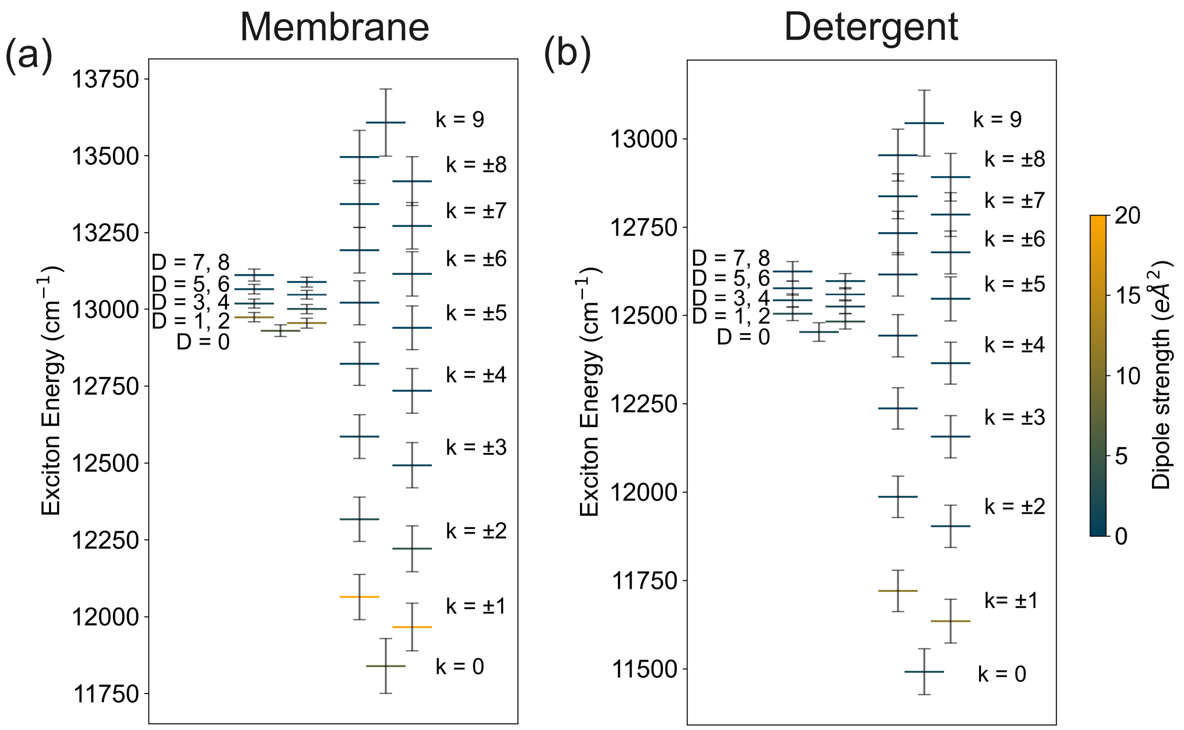

Figure S5: Average positions of the exciton energy levels of the B800 and B850 rings of (a) membrane embedded LH2 and (b) detergent isolated LH2 averaged over 10,000 realisations of static disorder. The disorder expected in each ring for each environment is given in Table S2. Standard deviations for each level are given as error bars.

Supporting References

[1] Mino Yang and Graham R Fleming. Influence of phonons on exciton transfer dynamics: comparison of the redfield, förster, and modified redfield equations. *Chem. Phys.*, 282(1):163–180, 2002.

[2] H. A. van der Vorst. Bi-cgstab: A fast and smoothly converging variant of bi-cg for the solution of nonsymmetric linear systems. *SIAM J. Sci. Comput.*, 13(2):631–644, 1992.

[3] Lorenzo Cupellini, Sandro Jurinovich, Marco Campetella, Stefano Caprasecca, Ciro A Guido, Sharon M Kelly, Alastair T Gardiner, Richard Cogdell, and Benedetta Mennucci. An ab initio description of the excitonic properties of lh2 and their temperature dependence. *J. Phys. Chem. B.*, 120(44):11348–11359, 2016.

[4] Felipe Cardoso Ramos, Michele Nottoli, Lorenzo Cupellini, and Benedetta Mennucci. The molecular mechanisms of light adaption in light-harvesting complexes of purple bacteria revealed by a multiscale modeling. *Chem. Sci.*, 10(42):9650–9662, 2019.

[5] Sergei Tretiak, Chris Middleton, Vladimir Chernyak, and Shaul Mukamel. Bacteriochlorophyll and carotenoid excitonic couplings in the lh2 system of purple bacteria. *J. Phys. Chem. B.*, 104(40):9540–9553, 2000.

[6] John I Ogren, Ashley L Tong, Samuel C Gordon, Aurélia Chenu, Yue Lu, Robert E Blankenship, Jianshu Cao, and Gabriela S Schlau-Cohen. Impact of the lipid bilayer on energy transfer kinetics in the photosynthetic protein lh2. *Chem. Sci.*, 9(12):3095–3104, 2018.

[7] Arvi Freiberg, Margus Rätsep, and Kõu Timpmann. A comparative spectroscopic and kinetic study of photoexcitations in detergent-isolated and membrane-embedded lh2 light-harvesting complexes. *Biochimica et Biophysica Acta (BBA)-Bioenergetics*, 1817(8):1471–1482, 2012.

[8] Ritesh Agarwal, Abbas H Rizvi, Bradley S Prall, John D Olsen, C Neil Hunter, and Graham R Fleming. Nature of disorder and inter-complex energy transfer in lh2 at room temperature: a three pulse photon echo peak shift study. *J. Phys. Chem. A.*, 106(33):7573–7578, 2002.

[9] Villy Sundström, Tõnu Pullerits, and Rienk van Grondelle. Photosynthetic light-harvesting: reconciling dynamics and structure of purple bacterial lh2 reveals function of photosynthetic unit, 1999.

[10] Kenneth Sauer, Richard J Cogdell, Steve M Prince, Andy Freer, Neil W Isaacs, and Hugo Scheer. Structure-based calculations of the optical spectra of the lh2 bacteriochlorophyll-protein complex from rhodopseudomonas acidophila. *Photochem. Photobiol.*, 64(3):564–576, 1996.

[11] Brent P Krueger, Gregory D Scholes, and Graham R Fleming. Calculation of couplings and energy-transfer pathways between the pigments of lh2 by the ab initio transition density cube method. *J. Phys. Chem. B.*, 102(27):5378–5386, 1998.

[12] Arvi Freiberg, Margus Rätsep, Kõu Timpmann, and Gediminas Trinkunas. Excitonic polarons in quasi-one-dimensional lh1 and lh2 bacteriochlorophyll a antenna aggregates from photosynthetic bacteria: A wavelength-dependent selective spectroscopy study. *Chem. Phys.*, 357(1-3):102–112, 2009.
