# Supplementary material for: Theory of photosynthetic membrane influence on B800-B850 energy transfer in the LH2 complex
Source: Biophys J. 2025 Jan 22;124(5):722–39. doi: 10.1016/j.bpj.2025.01.011 (PMC11897548; doi:10.1016/j.bpj.2025.01.011)
Supplement: Document S2. Article plus supporting material [file mmc2.pdf]

Biophysical Journal
Article

Biophysical Society

Theory of photosynthetic membrane influence on B800-B850 energy transfer in the LH2 complex

Chawntell Kulkarni,[1,*] Hallmann Óskar Gestsson,[2] Lorenzo Cupellini,[3] Benedetta Mennucci,[4] and Alexandra Olaya-Castro[5,*]

[1]Department of Physics and Astronomy, University College London, London, United Kingdom; [2]Department of Physics and Astronomy, University College London, London, United Kingdom; [3]Dipartimento di Chimica e Chimica Industriale, Università di Pisa, Pisa, Italy; [4]Dipartimento di Chimica e Chimica Industriale, Università di Pisa, Pisa, Italy; and [5]Department of Physics and Astronomy, University College London, London, United Kingdom

ABSTRACT Photosynthetic organisms rely on a network of light-harvesting protein-pigment complexes to efficiently absorb sunlight and transfer excitation energy to reaction center proteins where charge separation occurs. In photosynthetic purple bacteria, these complexes are embedded within the cell membrane, with lipid composition affecting complex clustering, thereby impacting inter-complex energy transfer. However, the impact of the lipid bilayer on intra-complex excitation dynamics is less understood. Recent experiments have addressed this question by comparing photo-excitation dynamics in detergent-isolated light-harvesting complex 2 (LH2) to LH2 complexes embedded in membrane discs mimicking the biological environment, revealing differences in spectra and energy-transfer rates. In this paper, we use available quantum chemical and spectroscopy data to develop a complementary theoretical study on the excitonic structure and intra-complex energy-transfer kinetics of the LH2 of photosynthetic purple bacteria *Rhodoblastus (Rbl.) acidophilus* (formerly *Rhodopseudomonas acidophila*) in two different conditions: the LH2 in a membrane environment and detergent-isolated LH2. We find that dark excitonic states, crucial for B800-B850 energy transfer within LH2, are more delocalized in the membrane model. Using nonperturbative and generalized Förster calculations, we show that such increased quantum delocalization results in a 30% faster B800 to B850 transfer rate in the membrane model, in agreement with experimental results. We identify the dominant energy-transfer pathways in each environment and demonstrate how differences in the B800 to B850 transfer rate arise from changes in LH2's electronic properties when embedded in the membrane. Furthermore, by accounting for the quasi-static variations of electronic excitation energies in the LH2, we show that the broadening of the distribution of the B800-B850 transfer rates is affected by the lipid composition. We argue that such variation in broadening could be a signature of a speed-accuracy trade-off, commonly seen in biological process.

SIGNIFICANCE Understanding the kinetics of energy transfer within photosynthetic light-harvesting complexes under conditions as close as possible to their biological environments will provide deeper insight into the biological mechanisms affecting their function. Experiments have shown that, for the LH2 complex of photosynthetic purple bacteria, a physiological membrane environment can enhance the efficiency of the key energy-transfer step within each complex compared to when the photosynthetic protein is isolated through chemical methods. We develop a comprehensive theoretical analysis that rationalizes these experimental observations and provides insight into quantum features and microscopic energy-transfer pathways that may be enhanced in the membrane environment, underpinning the increased energy-transfer rates.

INTRODUCTION

In purple nonsulfur bacteria, the initial steps of photosynthesis are carried out by a network of protein-pigment complexes that are embedded in the bacterial cell membrane (1). The network is built up of two types of complexes: the light-harvesting complex 2 (LH2) and LH1, which are responsible for the absorption and transfer of incident solar energy, and the reaction center (RC), which accepts excitation energy from the LH1 to facilitate transmembrane charge separation. Since the LH1 surrounds the RC, together they form the core light-harvesting complex (LH1-RC). Each LH1-RC is surrounded by several LH2 complexes, forming clusters on the cell membrane (2–4).

Submitted August 8, 2024, and accepted for publication January 17, 2025.

*Correspondence: chawntell.kulkarni.15@ucl.ac.uk or a.olaya@ucl.ac.uk

Editor: Abhishek Singharoy.

https://doi.org/10.1016/j.bpj.2025.01.011

© 2025 The Author(s). Published by Elsevier Inc. on behalf of Biophysical Society.
This is an open access article under the CC BY license (http://creativecommons.org/licenses/by/4.0/).

Here, we focus on the LH2 from the purple bacteria *Rhodoblastus (Rbl.) acidophilus*, which is composed of nine subunits that are arranged in a cyclic C9 symmetry (5,6). Each subunit consists of one $\alpha\beta$ heterodimer formed from two peptides (α and β), which bind three bacteriochlorophyll a chromophores (BChl a) and one carotenoid. BChl a absorbs light in the infrared region and is named according to the wavelength of light it approximately absorb at. Each subunit contains one B800 BChl and two B850 BChl a, labeled α and β according to the peptide it is ligated to. Due to the cyclic arrangement of the subunits in the LH2, two concentric rings of chromophores are formed: the B800 ring, which lies close to the inner cytoplasmic surface of the membrane, and the B850 ring, which lies close to the periplasmic surface. The transfer of excitation energy from chromophores in the B800 ring to the B850 ring is a key energy-transfer pathway within the LH2 (7–9).

Experimental studies focused on understanding the fundamental steps in photosynthetic light harvesting have contributed a vast amount of information on the structure and function of LH2 (1,10–14). Many of these studies isolate LH2 by solubilizing it in detergent, removing it from its native environment in the photosynthetic membrane. The impact of the membrane on the energy-transfer dynamics within LH2 remains an open question. Recently, experimental work has found differences in the spectra and energy transfer of detergent-isolated LH2 and membrane-embedded LH2 (15–18). With the existing comprehensive knowledge on the energy-transfer mechanism within detergent-isolated LH2, we have a benchmark to perform a systematic study of how energy transfer may be altered when LH2 is embedded in its native membrane environment.

The bacterial photosynthetic membrane is composed primarily of phospholipids, with different species of purple nonsulfur bacteria having varying lipid compositions (19,20). Lipids in the membrane mediate clustering of the LH2 complexes, with different lipid compositions resulting in different clustering tendencies (21,22). It has been suggested that the difference in organization of LH complexes can alter the efficiency of energy transfer from initial absorption by an LH2 complex to its arrival at the RC.

Live cells or sections of the native membrane have been studied but present difficulties due to the complex biological environment (23). Since whole cells are highly scattering, spectral signals are disturbed when using spectroscopic methods. To circumvent this issue, after isolating the LH2 with detergents, researchers then reconstitute LH2 into an artificial membrane and perform experiments on these samples (16,18,21,22,24,25). Initial studies comparing the spectroscopic properties of detergent-solubilized and membrane-reconstituted LH2 found little difference between the two, concluding that a single model should be sufficient to describe both scenarios (18).

In contrast, experiments comparing LH2 from *Rhodobacter (R.) sphaeroides* solubilized into detergent micelles to LH2 self-assembled into membrane vesicles found differences in the absorption spectra at room temperature (16,17). In the membrane vesicles, the B850 band of LH2 was broader and red shifted by 1.1 nm, and the Stokes shift between the absorption and fluorescence was greater in the membrane. Membrane vesicles typically contain multiple LH2 complexes, which, through their inter-complex interactions, can add another environmental contribution to the dynamics of a single LH2, leading to broadening in its spectra. Therefore, to isolate the membrane's effect on the complex, a single LH2 embedded in a membrane is ideal.

Ogren et al. embedded LH2 in a membrane nanodisc where each disc holds a single LH2, allowing a single complex to be separated and probed (15). The absorption spectra of a single LH2 complex in the membrane nanodisc also exhibited a slightly redshifted B850 absorption peak compared to detergent solubilized LH2.

In this work, we conduct a theoretical study of the impact of the membrane environment on energy transfer within the LH2 of *Rbl. acidophilus* to determine if the differences in spectra and energy-transfer times observed experimentally in *R. sphaeroides* hold across alternate species of purple bacteria and how these differences can be mapped down to microscopic changes in the energy-transfer pathways. Atomic-level calculations for electronic and environmental parameters are currently only available for membrane-embedded LH2 from *Rbl. acidophilus* (26). However, like *R. sphaeroides*, it contains nine subunits with cyclic C9 symmetry and produces similar linear absorption spectra (14,27) such that its structure is commonly used to model *R. sphaeroides* (28). Due to these structural and spectral similarities, we aim to see if the changes seen in the spectra and energy-transfer times of *R. sphaeroides* can be expected in *Rbl. acidophilus*. We compare two models of LH2, one based on experimental spectra of detergent solubilized LH2 (29) and the other describing LH2 embedded in a 1-palmitoyl-2-oleoyl-glycero-3-phosphocholine (POPC) membrane (26). We use two different spectral densities to describe the detergent and membrane environment and calculate energy-transfer rates within the LH2 using two different levels of theory: generalized Förster theory (GFT) (30), a perturbative method, and hierarchical equations of motion (HEOM), a numerically exact method. Due to the disordered nature of biological systems, each complex is perturbed differently by its local environment, creating slight variations in the electronic properties of each complex. Thus, we use many realizations of the electronic parameters to calculate inter-complex energy-transfer rates and exciton properties and analyze the specific form of their statistical distribution to see if they reveal anything about the membrane's influence on energy-transfer dynamics within the LH2. We compare the exciton delocalization for detergent-isolated LH2 and membrane-embedded LH2 using the inverse participation ratio as a measure. Using GFT and

HEOM, we calculate the B800 to B850 energy-transfer rate distribution for both models and consider the B800 and B850 exciton levels that form the dominating energy-transfer pathways in each environment.

METHODS

Hamiltonian

To model the LH2 complex, we divide the total system Hamiltonian into the system, the environment, and the interaction between the two:

$$\widehat{H} = \widehat{H}_{\mathrm{S}} + \widehat{H}_{\mathrm{B}} + \widehat{H}_{\mathrm{SB}}. \quad (1)$$

Here, $\widehat{H}_{\mathrm{S}}$ represents the electronic degrees of freedom of the N chromophores within the LH2 and is given by a Frenkel exciton Hamiltonian (31), where each chromophore site is treated as a two-level system (we have $\hbar = 1$ throughout),

$$\widehat{H}_{\mathrm{S}} = \sum_{i}^{N} E_i |i\rangle\langle i| + \sum_{i,j<i}^{N} V_{ij}(|i\rangle\langle j| + |j\rangle\langle i|), \quad (2)$$

where $|i\rangle$ is an excited state localized on site i. $E_i = \varepsilon_i + \lambda_i$ is the transition energy from ground to excited state of site i termed the site energy and is the sum of ε_i, the bare electronic energy in the absence of phonons, and λ_i the reorganization energy. $\lambda_i = \pi^{-1}\int_0^\infty d\omega\, J_i(\omega)/\omega$ is the energy the bath must dissipate to relax to the new equilibrium in the excited state $|i\rangle$, which can be obtained by integrating over the spectral density $J_i(\omega)$. The microscopic origin of λ_i is due to the excited state potential energy surface being displaced relative to the ground state (32). V_{ij} is the electronic coupling between the Q_y transition dipole moments at sites i and j. We denote $|\alpha\rangle$, the eigenstates of $\widehat{H}_s$ with energy E_α, i.e., $\widehat{H}_s = E_\alpha|\alpha\rangle$, which are collective electronic states, or excitons, delocalized across all chromophores, i.e., $|\alpha\rangle = \sum_i C_i^\alpha \Big| i\rangle$.

Site energies and nearest-neighbor electronic couplings for the membrane and detergent Hamiltonians are given in Table 1. For the detergent Hamiltonian, interchromophore electronic couplings are calculated using the dipole-dipole approximation,

$$V_{ij}^{\mathrm{dipole}} = C\frac{\widehat{\mathbf{d}}_i \cdot \widehat{\mathbf{d}}_j - 3\left(\widehat{\mathbf{r}}_{ij} \cdot \widehat{\mathbf{d}}_i\right)\left(\widehat{\mathbf{r}}_{ij} \cdot \widehat{\mathbf{d}}_j\right)}{\left|r_{ij}\right|^3}, \quad (3)$$

where C is a constant accounting for the dipole strength, $\widehat{\mathbf{d}}_i$ is the transition dipole unit vector at site i, $\widehat{\mathbf{r}}_{ij}$ is the unit vector pointing from the position of site i to site j, and r_{ij} is the distance between sites i and j. The site coordinates and transition dipole moments are taken from the crystal structure of LH2 from *Rbl. acidophilus* (5) and C is taken to be 230,000 Åcm^{-1} for the B800 sites and 348,000 Åcm^{-1} for the B850 sites, chosen to reproduce energies of the excitonic states. Additionally, these values of C produce couplings that agree with more sophisticated transition density cube methods used to determine electronic couplings in the LH2 (33,34). For nearest-neighbor electronic couplings in the B850 ring, the dipole-dipole approximation no longer holds due to the proximity of the chromophores; hence, couplings were taken from literature where they are fitted to reproduce experimental spectra (29). The electronic parameters for the membrane Hamiltonian were calculated using quantum chemical methods that account for the mutual polarization between the lipid-protein environment and the chromophores (26,35). Site energies and couplings are averaged over a trajectory of the LH2 in a lipid environment using molecular-dynamics simulations. The site energies and nearest-neighbor couplings of the B800 and B850 chromophores are taken from (26) and are given in Table 1.

To select the Hamiltonian parameters used to represent LH2 in each environment, we conducted a thorough review of the different parameter sets reported in the literature for both the LH2 in detergent and LH2 in membrane, as detailed in the supporting material. For the membrane-embedded LH2, to the best of our knowledge, the only reliable parameters available

TABLE 1 Site energies, nearest-neighbor electronic couplings, and environmental parameters of the chromophore sites in the LH2 from *Rbl*

		Membrane POPC	Membrane DOPC	Detergent
Site energy	B800	13,021	13,783	12,540
	B850α	12,799	13,527	12,390
	B850β	12,806	13,556	12,390
B800 couplings	V_{B800}	−33	−34	−19
B850 couplings	$V_{\alpha1\beta1}$	339	298	315
	$V_{\alpha2\beta1}$	317	266	245
B800 to B850 couplings	$V_{\mathrm{B800},\alpha2}$	42	38	32
Reorganization energy	λ_{B800}	40	40	35
	λ_{B850}	140	140	160
Cutoff frequency	Ω_{B800}	100	100	35
	Ω_{B850}	100	100	53
Static disorder	σ_{B800}	40	40	50
	σ_{B850}	270	270	220

Interchromophore electronic couplings are illustrated in Fig. 1 *b. Acidophilus* for the membrane and detergent models given in units of cm^{-1}. Membrane POPC parameters were taken from (26), DOPC membrane electronic parameters were taken from (36), and detergent parameters for the B850 ring were taken from (29).

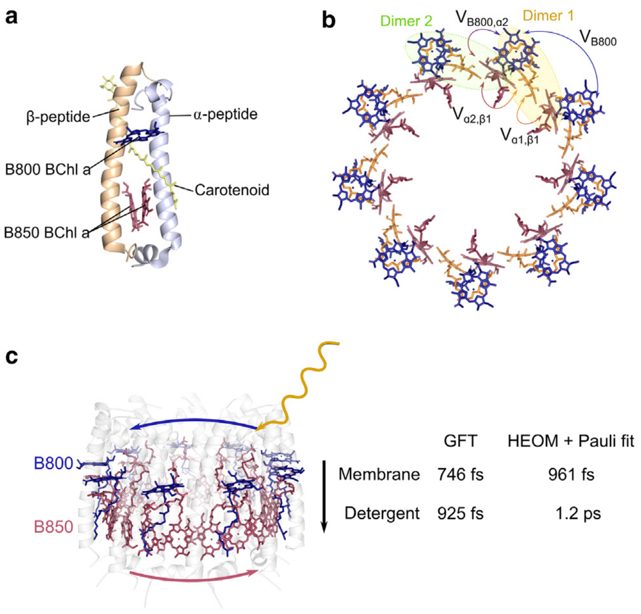

FIGURE 1 LH2 molecular structure, electronic interactions and energy transfer rates. (*a*) Subunit of the LH2 of *Rbl. acidophilus* (PDB: 1NKZ) (5) consisting of an $\alpha\beta$-heterodimer, one B800, and two B850 bacteriochlorophyll a chromophores and one carotenoid. (*b*) Top view of the chromophores in the LH2, B850α chromophores in red and B850β chromophores in orange. Nearest-neighbor chromophores couplings are labeled. (*c*) Side view of the LH2 showing the arrangement of the B800 ring (*blue*) and B850 ring (*red*) of chromophores. Following the absorption of solar energy by chromophores in the B800 ring, energy is transferred to the B850 ring. Timescales given for inter-ring transfer are computed using GFT and HEOM.

are those reported by some of us (26,36). For the LH2 in detergent, several parameter sets are reported (10,29,33,37). We computed linear spectra for the various parameter sets (Fig. S4) and compared predictions of the key spectral changes from detergent to membrane to what is observed in experiment, namely the redshift of the B850 absorption peak, which has been argued to be associated with changes in the electronic structure of the LH2. For all parameter sets, we find that the qualitative spectral differences between detergent and membrane hold in each case. However, the size of the redshift predicted by each parameter set varies, resulting in different B800 to B850 energy-transfer rate predictions (Table S1). We have selected the detergent parameters that predict a redshift that best compares quantitatively with the spectral changes observed in experiments that have motivated our study (15). The selected parameters lead to B800 to B850 transfer-rate predictions that compare well with the experimental observations (15) as shown in the supporting material.

The environment, H_{B}, corresponds to the intermolecular vibrations of the chromophores along with the motion of the proteins and is modeled as a bath of quantized harmonic oscillators (vibrational modes),

$$\widehat{H}_{\mathrm{B}} = \sum_{i,k}\omega_{i,k}\left(\widehat{b}^{\dagger}_{i,k}\widehat{b}_{i,k}+\frac{1}{2}\right), \tag{4}$$

where $b^{\dagger}_{i,k}$ and $b_{i,k}$ are bosonic creation and annihilation operators of frequency modes $\omega_{i,k}$ satisfying commutation relations $[b_{i,k}, b^{\dagger}_{j,k'}] = \delta_{i,j}\delta_{k,k'}$ (38). Each site is linearly coupled to an environment displacement mode such that the system-environment interaction is of the form

$$\widehat{H}_{\mathrm{SB}} = \sum_{i,k} g_{i,k}\left(\widehat{b}_{i,k}+\widehat{b}^{\dagger}_{i,k}\right)|i\rangle\langle i| = \sum_{i}\widehat{B}_i|i\rangle\langle i|, \tag{5}$$

where $g_{i,k}$ is the interaction strength.

Influence of the environment on the system dynamics may be described fully by the system-bath correlation function

$$C_i(t) = \langle \widehat{B}_i(t)\widehat{B}_j(0)\rangle_B \tag{6}$$

$$= \frac{1}{\pi}\int_0^{\infty} d\omega J_i(\omega)\left(\coth\left(\frac{\beta\omega}{2}\right)\cos(\omega t) - i\sin(\omega t)\right), \tag{7}$$

where $\beta = \frac{1}{k_{\mathrm{B}}T}$.

Within each band of LH2, we assume that local electronic-vibrational interactions are identical such that all sites are characterized by the same spectral density which takes the Drude-Lorentz form,

$$J_i(\omega) = 2\lambda_i\gamma_i\frac{\omega}{\omega^2+\gamma_i^2}, \tag{8}$$

where γ_i is the cutoff frequency corresponding to the bath relaxation rate. For a Drude-Lorentz spectral density, the bath correlation function may be expressed as an exponential series (32)

$$C_i(t) = \sum_k c_{k,i}e^{-\nu_{k,i}t}, \tag{9}$$

where the coefficients and rates that enter the expansion are obtained using the Matsubara expansion method, $c_{0,i} = \lambda_i\gamma_i\left(\cot\left(\frac{\beta\gamma_i}{2}\right) - i\right)$, $\nu_{0,i} = \gamma_i$, $c_{k,i} = \frac{4\lambda_i\gamma_i}{\beta}\frac{\nu_k}{\nu_k^2-\gamma_i^2}$, and $\nu_{k,i} = \nu_k$, where $\nu_k = \frac{2\pi k}{\beta}$ are the Matsubara frequencies with $k = 1, 2, 3\ldots$. The environmental parameters introduced here, λ_i and γ_i, are given for membrane-embedded and detergent-isolated LH2 in Table 1.

Static disorder

In the previous section, fixed electronic parameters were given for the chromophore sites in the LH2. However, owing to the dynamic nature of the biological environment, slow conformational motions of the proteins lead to random shifts in the electronic parameters of the chromophores (1). Stochastic fluctuations in the local environment of the chromophores create shifts in their site energies, whereas changes in the orientation and position of the chromophore transition dipole moments alter interchromophore couplings (39). Since these changes are slow compared to energy-transfer timescales, they can be accounted for by taking an ensemble average over many realizations of the electronic parameters.

Various models of static disorder have been suggested for the LH2, including Gaussian site energy disorder and elliptical disorder (40–42). Although elliptical disorder has been shown to describe low-temperature single-molecule spectra (42), Gaussian site energy disorder describes ensemble spectra at both low (40) and room temperature (41,43,44) well. Additionally, for the lowest B850 exciton, $k = 0$, which contributes to the B850 absorption band, Gaussian site energy disorder produces a dipole strength that is comparable with experimental superradiance values (44,45), whereas elliptical disorder underestimates it (43). As we are interested in modeling the LH2 at physiological temperatures, Gaussian site energy disorder is an appropriate model of disorder to use. Therefore, we account for static disorder by adding an offset $\delta_i^r \in \{\delta_i\}_r$ to the site energies of the system Hamiltonian in the chromophore site basis,

$$\widehat{H}^r_{\mathrm{S}} = \sum_i^N\left(E_i+\delta_i^r\right)|i\rangle\langle i| + \sum_{i,j<i}^N V_{ij}(|i\rangle\langle j| + |j\rangle\langle i|), \tag{10}$$

where r labels a particular realization. Each δ_i^r is randomly sampled from a Gaussian distribution centered at zero, whose standard deviation, σ, corresponds to the level of static disorder. Hence, excitonic energies and exciton delocalization are different for each realization. To account for the effects of static disorder on the system, calculations of observables are averaged over several thousands of realizations of static disorder until we achieve convergence. Static disorder standard deviations for the B800 sites and B850 sites in detergent and membrane are given in Table 1.

l1 norm of coherence

Due to strong interchromophore electronic couplings in the B850 ring, an excitation in the ring manifests as a delocalized exciton state spread across multiple chromophore sites. To quantify the delocalization of an exciton state $|\alpha\rangle$, we use two measures: the l1 norm of coherence (46) and the more common inverse participation ratio. This allows us to analyze whether different quantifiers of exciton delocalization lead to the same conclusions.

The l1 norm of coherence, denoted as C_{l1} (46), is a measure of coherence based on distance measures and represents the distance of the density matrix associated to $\langle\alpha|$, i.e., $\widehat{\rho}^{\alpha} = |\alpha\rangle\langle\alpha|$ to the set of incoherent quantum states in the reference basis $\{|i\rangle\}$. $C_{l1}(\widehat{\rho}^{\alpha})$ is then given by

$$C_{l1}(\widehat{\rho}^{\alpha}) = \sum_{i,j\neq i}\left|\widehat{\rho}^{\alpha}_{i,j}\right| = \sum_{i,j\neq i}\left|C_i^{\alpha}\left(C_j^{\alpha}\right)^*\right|, \tag{11}$$

where $C_i^{\alpha} = \langle i|\alpha$ is the amplitude of the excited state of chromophore i in the exciton eigenstate $|\alpha\rangle$. Under incoherent processes, C_{l1} does not increase and therefore it provides an appropriate quantifier of coherence (47).

A more common measure of exciton delocalization is the inverse participation ratio (IPR), which is given by,

$$\mathrm{IPR}_{\alpha} = \frac{1}{\sum_i^N\left|C_i^{\alpha}\right|^4}, \tag{12}$$

where C_i^{α} is as defined above. The IPR represents how many chromophores an exciton state $|\alpha\rangle$ is extended over. For example, for a localized exciton, IPR $= 1$, whereas, for a completely delocalized exciton, IPR $= N$, where N is the number of chromophores in the ring.

HEOM

To quantify energy-transfer rates within the LH2, we apply the HEOM (48,49) to compute the quantum dynamics for the full 27-site model of LH2 that includes both the B800 and B850 and interactions among them to predict linear spectra and estimate transfer rates. The HEOM can yield exact quantitative results for the electronic dynamics provided that system-environment correlation functions are represented by an exponential series expansion as in Eq. 7.

The HEOM is of the form

$$\hat{\rho}_{\boldsymbol{n}} = \left(\mathcal{L} - \Xi - \sum_{k,i} n_{k,i}\nu_{k,i}\right)\hat{\rho}_{\boldsymbol{n}} - i\sum_{k,i}\left(\mathcal{L}^{-}_{k,i}\hat{\rho}_{\boldsymbol{n}^{-}_{k,i}} + \mathcal{L}^{+}_{k,i}\hat{\rho}_{\boldsymbol{n}^{+}_{k,i}}\right), \tag{13}$$

where n is a multi-index consisting of discrete integers $n_{k,i}$. An auxiliary density operator (ADO) $\hat{\rho}_{\boldsymbol{n}}$ is said to belong the n-th tier of the hierarchy if $\sum_{k,i} n_{k,i} = n$. The reduced density matrix of the system is identified as ρ_0. The hierarchy in Eq. 13 is formalized in terms of super-operators such that, for an arbitrary system operator $\hat{A}$,we may write $\hat{A}^{\times}$ and $\hat{A}^{\circ}$, which denote super-operators whose action onto a system space operator $\hat{B}$ is given by $\hat{A}^{\times}\hat{B} = [\hat{A},\hat{B}]$ and $\hat{A}^{\circ}\hat{B} = \{\hat{A},\hat{B}\}$. We have

$$\mathcal{L} = -i\hat{H}_{\mathrm{S}}^{\times}, \tag{14}$$

$$\mathcal{L}^{-}_{k,i} = \mathrm{Re}(c_{k,i})\hat{n}_i^{\times} + i\mathrm{Im}(c_{k,i})\hat{n}_i^{\circ}, \tag{15}$$

$$\mathcal{L}^{+}_{k,i} = \hat{n}_i^{\times}. \tag{16}$$

We truncate the hierarchy by setting all ADOs beyond a pre-set hierarchy tier to zero. The truncation tier L is simultaneously set to be large enough such that numerical results have converged and small enough so that the simulation will run in a reasonable amount of time. The Matsubara series is truncated as well by approximating $e^{-\nu_k t} \approx \frac{1}{\nu_k}\delta(t)$ for all $k \geq M$, where M is another pre-set threshold chosen similarly to L. These approximated terms for the series expansion are then described by the terminator term $\Xi = \sum_m \left(\frac{2\lambda_m}{\beta\gamma_m}\left(1 - \frac{\beta\gamma_m}{2}\cot\left(\frac{\beta\gamma_m}{2}\right)\right) - \sum_{k=1}^{M}\frac{c_{k,m}}{\nu_k}\right)\hat{n}_m^{\times}\hat{n}_m^{\times}$ (50). We furthermore improve convergence of the HEOM results by applying the scaling procedure developed by Shi and co-workers (51).

Exact ring population dynamics and its fit to a Pauli master equation

To estimate B800 to B850 energy-transfer rates based on the HEOM dynamics, we take our initial state to be the Boltzmann distribution for the B800 eigenstates, i.e., $\hat{\rho}(0) = \frac{e^{-\beta\hat{H}_{\mathrm{B800}}}}{\mathrm{Tr}(e^{-\beta\hat{H}_{\mathrm{B800}}})}$, which is then propagated in time as per the HEOM in Eq. 13. We define the total B800 population dynamics as $P_{B800}(t) = \sum_{\alpha\in B800}\langle\alpha|\hat{\rho}(t)|\alpha\rangle$ with $|\alpha\rangle$ the exciton eigenstates of $\hat{H}_{B800}$, and similarly for the total B850 population dynamics, $P_{B850}(t)$. To estimate the transfer rates from B800 to B850, once a steady state is reached, we fit P_{B800} and P_{B850} to a Pauli master equation of the form

$$\partial_t\begin{bmatrix}P_{\mathrm{B800}}\\P_{\mathrm{B850}}\end{bmatrix} = \begin{bmatrix}-k_{\mathrm{down}} & k_{\mathrm{up}}\\k_{\mathrm{up}} & -k_{\mathrm{down}}\end{bmatrix}\begin{bmatrix}P_{\mathrm{B800}}\\P_{\mathrm{B850}}\end{bmatrix}, \tag{17}$$

where k_{up} and k_{down} are uphill and downhill decay rates corresponding to the B850 → B800 and B800 → B850 transfer process, respectively. We can solve for P_{B800} by using the fact that $P_{\mathrm{B800}}(t) + P_{\mathrm{B850}}(t) = 1$ such that the B800 population dynamics is of the form

$$P_{\mathrm{B800}}(t) = \frac{k_{\mathrm{up}} + k_{\mathrm{down}}e^{-\left(k_{\mathrm{up}}+k_{\mathrm{down}}\right)t}}{k_{\mathrm{up}} + k_{\mathrm{down}}}, \tag{18}$$

where the k_{down} and k_{up} are numerically determined from a fit to HEOM-simulated population dynamics. This procedure allows estimation of rates that are qualitatively comparable to GFT rates but we do not expect a full quantitative agreement as we are effectively mapping the kinetics of transfer to a two-state system, whereas GFT rates consider multiple parallel processes of exciton to exciton transfer. We will indeed show the qualitative agreement between HEOM and GFT rates and therefore find that the results from the exact treatment support the insight gained from GFT.

Linear spectra

Linear absorption spectra are computed using

$$\alpha_{\mathrm{A}}(\omega) = \mathrm{Re}\left[\sum_{p=x,y,z}\int_0^{\infty}\mathrm{d}t\langle\hat{\mu}_p(t)\hat{\mu}_p(0)\rangle_{\rho_0}e^{i\omega t}\right], \tag{19}$$

where the initial state of the system is the ground state $\rho_0 = |0\rangle\langle 0|$ and $\hat{\mu}_p(t)$ is the Heisenberg picture dipole operator corresponding to the p direction. The dipole operators are of the form

$$\hat{\mu}_p = \sum_i d_{i,p}|i\rangle\langle 0| + \mathrm{h.c.}, \tag{20}$$

where $d_{i,p}$ is the p component of the dipole at site i.

Linear fluorescence spectra are computed using

$$I_{\mathrm{D}}(\omega) = \mathrm{Re}\left[\sum_{p=x,y,z}\int_0^{\infty}\mathrm{d}t\langle\hat{\mu}_p(t)\hat{\mu}_p(0)\rangle_{\rho_{\mathrm{th}}}e^{i\omega t}\right], \tag{21}$$

where the initial state of the system is the thermal steady state of the system. We determine ρ_{th} iteratively via the biconjugate gradient stabilized method (52) with an initial guess given by the Boltzmann distribution $\rho(0) = \frac{e^{-\beta H_{\mathrm{B800}}}}{\mathrm{Tr}(e^{-\beta H_{\mathrm{B800}}})}\oplus\mathbb{I}_{B850}$, where $\mathbb{I}_{B850}$ is the identity for the single excitation subspace of the B850 ring.

GFT

In addition to HEOM, we use GFT to calculate the B800 to B850 energy-transfer rate. By doing so, we can confirm that our results hold qualitatively at different levels of theory and are not dependent on the approximations made in GFT. Additionally, GFT is a less computationally expensive method that allows the computation of more realizations of static disorder within a reasonable time frame.

GFT describes exciton energy transfer from a donor aggregate to an acceptor aggregate that are weakly coupled to one another (53–55). It is assumed that, within each aggregate, electronic couplings are strong such that an excitation forms a delocalized exciton state. In the LH2, the donor and acceptor aggregates correspond to the B800 and B850 rings. Strong interchromophore couplings in each ring allow for an excitation to be delocalized across the ring instead of being confined to a single chromophore site.

To model B800 to B850 energy transfer, it is assumed that, after an electronic transition in the B800 ring, thermal relaxation occurs on a shorter timescale than energy transfer, such that transfer to B850 occurs from a thermally populated B800 state. Thus, the B800 to B850 energy-transfer rate is given by (53)

$$K_{\mathrm{GFT}} = \sum_{\alpha,\beta} P_\alpha k_{\alpha\beta}, \tag{22}$$

where α labels a donor exciton, β labels an acceptor exciton, P_α is the thermal population of the donor state, and k is the exciton transfer rate from α to β, which is given by the product of the square magnitude of the exciton coupling and the exciton spectral overlap,

$$k_{\alpha\beta} = \left|V_{\alpha\beta}\right|^2 O_{\alpha\beta}. \tag{23}$$

V $_{\alpha\beta}$ is given by (55)

$$V_{\alpha\beta} = \sum_{i\in D, j\in A} C_i^\alpha C_j^{\beta*} V_{ij}, \tag{24}$$

where $C_i^\alpha = \langle i|\alpha$ is the amplitude coefficient of site i in the donor exciton eigenstate.

$O_{\alpha\beta}$ is the spectral overlap between the donor fluorescence lineshape $\tilde{D}_\alpha(\omega)$ and acceptor absorption lineshape $D_\beta(\omega)$ given by

$$O_{\alpha\beta}(\omega) = \frac{1}{2\pi}\int_{-\infty}^{\infty} \mathrm{d}\omega \tilde{D}_\alpha(\omega) D_\beta(\omega). \tag{25}$$

The form of the lineshape functions may be obtained using perturbative theories as given in the following section.

Lineshape theory

To determine the lineshapes, we follow the method outlined by Renger (56) where the second-order cumulant expansion is used to derive an equation of motion of the reduced system density matrix. This yields lineshape functions of the form

$$\tilde{D}_\alpha(\omega) = 2\mathrm{Re}\int_0^\infty \mathrm{d}t\, e^{i\omega t} e^{-i\left(\omega_\alpha - \lambda_{\alpha\alpha,\alpha\alpha}\right)t - g^*_{\alpha\alpha,\alpha\alpha}(t) - t/\tau_\alpha}, \tag{26}$$

$$D_\beta(\omega) = 2\mathrm{Re}\int_0^\infty \mathrm{d}t\, e^{i\omega t} e^{-i\left(\omega_\beta - \lambda_{\beta\beta,\beta\beta}\right)t - g_{\beta\beta,\beta\beta}(t) - t/\tau_\beta}, \tag{27}$$

for which $O_{\alpha\beta}$ may be written as

$$O_{\alpha\beta}(\omega) = 2\mathrm{Re}\int_0^\infty \mathrm{d}t e^{i\omega_{\alpha\beta}t} e^{-i\left(\lambda_{\alpha\alpha,\alpha\alpha}+\lambda_{\beta\beta,\beta\beta}\right)t} \times e^{-\left(g_{\alpha\alpha,\alpha\alpha}(t)+g_{\beta\beta,\beta\beta}(t)\right)} e^{-\left(1/\tau_\alpha + 1/\tau_\beta\right)t}, \tag{28}$$

where ω_α is the energy of exciton α, $\lambda_{\alpha\beta,\gamma\delta} = \sum_i (C_i^\alpha)^* C_i^\beta (C_i^\gamma)^* C_i^\delta \lambda_i$ is the exciton reorganization energy, $g_{\alpha\beta,\gamma\delta}(t) = \sum_i (C_i^\alpha)^* C_i^\beta (C_i^\gamma)^* C_i^\delta g_i(t)$ is the exciton line broadening function, and τ_α is the lifetime of exciton α. The exciton lifetimes are approximated using modified Redfield theory (57) as outlined in the supporting material. $g_i(t)$ is the site line-broadening function, which, for the bath correlation function we consider (Eq. 9), may be written as

$$g_i(t) = \frac{c_{0,i}}{\gamma_i^2}\left(e^{-\gamma_i t} + \gamma_i t - 1\right) + \sum_{k=1} \frac{c_{k,i}}{\nu_k^2}\left(e^{-\nu_k t} + \nu_k t - 1\right), \tag{29}$$

The Matsubara summation terms labeled by k are low-temperature corrections to the exponential expansion of the bath correlation function. Since we are interested in the function of LH2 in a physiological environment, our calculations are at 300K, where the Matsubara terms are less important. We truncate the summation at $k = 1$, as the correlation function C_i does not change when including higher-order terms.

Aside from computing energy-transfer rates, the lineshapes in Eqs. 27 and 26 are also used to compute linear spectra. The linear absorption and fluorescence spectra of the respective B800 and B850 rings can be obtained using their relationship to $D_\beta(\omega)$ and $\tilde{D}_\alpha(\omega)$ (55),

$$\alpha_A(\omega) \propto \sum_\beta \left|\vec{\mu}_\beta\right|^2 D_\beta(\omega), \tag{30}$$

$$I_D(\omega) \propto \sum_\alpha P_\alpha \left|\vec{\mu}_\alpha\right|^2 \tilde{D}_\alpha(\omega), \tag{31}$$

where $\left|\vec{\mu}_\alpha\right|$ is the transition dipole strength of exciton α given by $\left|\vec{\mu}_\alpha\right|^2 = \left|\sum_i C_i^\alpha \vec{\mu}_i\right|^2$ and $\vec{\mu}_i$ is the transition dipole moment at chromophore site i.

RESULTS

We begin by examining properties of the excitons that have been well documented by previous theoretical and experimental work on the LH2 and see how they are altered for LH2 embedded in a lipid membrane environment. Motivated by experiment, we focus on comparing POPC membrane LH2 to detergent LH2, but similar conclusions apply to 1,2-dioleoyl-*sn*-glycero-3-phosphocholine (DOPC) membrane. We finally compare B800 to B850 transfer rates computed using GFT and HEOM in each environment and determine the main energy-transfer pathways that contribute to the transfer rate to see how they change from membrane to detergent.

Exciton energy vs. static disorder

The B800 and B850 Hamiltonians were diagonalized to obtain B800 exciton energies and B850 exciton energies, respectively. In the absence of static disorder, the B800 exciton manifold consists of one low-lying energy level, followed by four pairs of doubly degenerate levels. In the B850 manifold, the lower-energy exciton levels have a similar structure, consisting of one low-energy level followed by four doubly degenerate levels. The higher energy levels, B850* (7,8), consist of four doubly degenerate levels followed by a single highest-energy level.

Fig. 2 *a* and *b* give the exciton energy levels of the B850 ring as a function of static disorder averaged over 10,000 realizations for membrane and detergent LH2, respectively. As static disorder increases, the degeneracy of the exciton levels is lifted and the average energy levels begin to diverge. Following the inclusion of static disorder, the k quantum number for each eigenstate is no longer well defined. Here, we use k simply for ease of labeling, with negative k values referring to the lower energy level.

Exciton transition dipole strength vs. static disorder

Through the interaction of $\vec{\mu}_\alpha$ with an electromagnetic field, an optical transition from the ground state to the excited state, or vice versa, is possible. Thus, $|\vec{\mu}_\alpha|^2$ can tell us if a transition to a given exciton state is optically allowed, as it defines the strength of the interaction between $\vec{\mu}_\alpha$ and the electromagnetic field.

Fig. 2 *c* and *d* gives $\vec{\mu}_\alpha$ for the five lowest-lying levels in the B850 ring for increasing static disorder averaged over 10,000 realizations for membrane and detergent LH2, respectively. Without accounting for static disorder, the $k = \pm 1$ states of the B850 ring are the only bright states; i.e., almost all the transition dipole strength in the system is associated with them. As static disorder increases, the transition dipole strength is redistributed to neighboring exciton states that are close in energy to $k = \pm 1$, namely $k = 0, \pm 2$. The $k = \pm 1$ states still retain a majority of the transition dipole strength when accounting for static disorder, making them most important for energy transfer to the B850 ring via optical transitions.

Exciton energy levels and dipole strengths at defined static disorder

Fig. 3 shows the average positions of the B800 and B850 exciton energy levels calculated using 10,000 realizations of static disorder for membrane-embedded LH2 and detergent-isolated LH2. The average energy levels plotted in Fig. 3 are plotted with error bars representing the standard deviation in Fig. S5, with the standard deviations provided in Tables S2 and S3.

Due to the differences in the average excitonic structure in membrane and detergent LH2, the B800 excitons overlap spectrally with different B850 excitons in each environment, which impacts the key B800 to B850 energy-transfer pathways. For the membrane, there is a greater overlap on average between the B800 states and the dark B850* states, whereas, for detergent, the overlap is with lower-energy B850 states. The energy-transfer pathway that dominates the B800 to B850 transfer in each environment as determined by GFT is shown by the red arrows in Fig. 3. Differences in energy-transfer pathways can result in differences in the overall B800 to B850 transfer rate.

Exciton delocalization

We can examine differences in the delocalization of excitons in membrane and detergent LH2 by calculating the C_{l1} for excitons localized on each respective ring, where excitations are understood to be superpositions of excited states localized on single sites. We additionally calculate the IPR of the excitons and compare the two measures of delocalization.

At zero static disorder, excitons have the same IPR in all environments, apart from a small 6% increase in POPC membrane and DOPC membrane for four B850 levels, $k = \pm 4$, and $k = \pm 5$ relative to the same excitons in detergent. Noticeable differences start to emerge when the IPR is calculated at

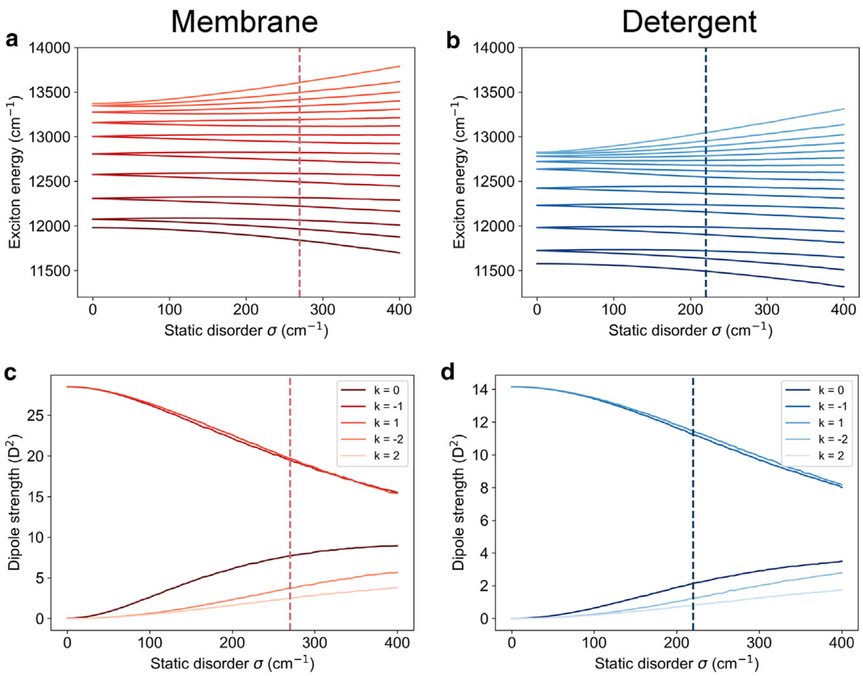

FIGURE 2 Exciton energy and exciton dipole strength vs. static disorder. (*a and b*) Exciton energy levels of the B850 ring as a function of static disorder and (*c and d*) exciton transition dipole strength for the lowest five levels of the B850 ring averaged over 10,000 realizations for detergent and membrane, respectively. The level of static disorder expected in the B850 ring is given by the red dotted line for membrane-embedded LH2 and by the blue dotted line for detergent-isolated LH2.

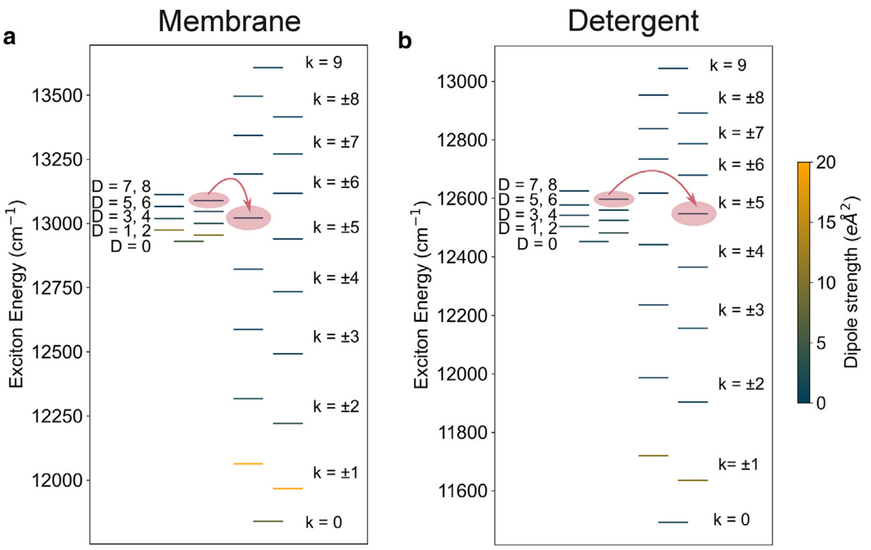

FIGURE 3 Disorder averaged exciton energy levels. Average positions of the exciton energy levels of the B800 and B850 rings of (*a*) POPC membrane-embedded LH2 and (*b*) detergent-isolated LH2 averaged over 10,000 realizations of static disorder. The disorder expected in each ring for each environment is given in Table 1. Red arrows indicate the dominating B800 to B850 energy-transfer pathways, determined using GFT.

the level of static disorder expected in each ring. Tables 2 and 3 gives C_{l1} and the IPR averaged over 10,000 realizations of static disorder for B850 and B800 excitons, respectively. An overall decrease in the delocalization is seen across all excitons. This is because static disorder creates random shifts in the electronic parameters of the chromophores such that their site energies are no longer identical, reducing the symmetry of the system, which tends to localize the excitons. However, in each environment, the localizing effect of static disorder perturbs each exciton differently.

C_{l1} is a proper measure of coherence based on distance measures, and hence it provides a more reliable value to compare the delocalization of different states. For example, take the B850 states k $= -3$ and k $= +2$; the IPR is equal for these states, yet C_{l1} reveals that they have different delocalization. For other states, the IPR predicts different delocalization when C_{l1} shows that those states have identical delocalization. Thus, the IPR can be misleading when a comparison of state delocalization is desired.

TABLE 2 The *l*1 norm of coherence and IPR of the B850 excitons in POPC membrane-embedded LH2, DOPC membrane-embedded LH2, and detergent-isolated LH2 averaged over 10,000 realizations of disorder listed from highest to lowest energy

	C_{l1}			IPR		
Exciton	POPC Membrane	DOPC Membrane	Detergent	POPC Membrane	DOPC Membrane	Detergent
k = 9	4	3	3	3	2	2
k = +8	5	4	3	3	3	3
k = −8	6	5	4	4	3	3
k = +7	7	6	5	5	4	3
k = −7	9	8	6	5	4	4
k = +6	9	8	7	6	5	4
k = −6	11	9	8	7	6	5
k = +5	11	10	9	7	6	5
k = −5	12	11	10	8	7	6
Average	8	7	6	5	4	4
k = +4	12	11	11	8	7	7
k = −4	12	11	11	9	8	9
k = +3	12	12	12	9	8	9
k = −3	12	11	12	9	8	9
k = +2	13	12	13	9	8	10
k = −2	12	11	12	8	8	9
k = +1	12	12	13	8	8	10
k = −1	10	10	11	7	6	8
k = 0	10	9	13	6	5	9
Average	12	11	12	8	7	9
Average	10	9	9	7	6	6

A line dividing the states in half separates what we describe as the high-energy manifold from the low-energy manifold.

TABLE 3 The $l1$ norm of coherence and IPR of the B800 excitons in POPC membrane-embedded LH2, DOPC membrane-embedded LH2, and detergent-isolated LH2 averaged over 10,000 realizations of disorder listed from highest to lowest energy

	C_{l1}			IPR		
Exciton	POPC Membrane	DOPC Membrane	Detergent	POPC Membrane	DOPC Membrane	Detergent
D = 8	3	4	2	3	3	2
D = 7	5	5	2	4	4	2
D = 6	5	5	3	4	4	2
D = 5	6	6	3	5	5	3
D = 4	6	6	4	5	5	3
D = 3	5	5	4	4	5	3
D = 2	6	6	4	4	4	3
D = 1	4	4	3	3	3	2
D = 0	4	4	3	3	3	2
Average	5	5	3	4	4	2

Comparing the average C_{l1} of the B800 excitons, $(\overline{C_{l1}(\rho^\alpha)} = \frac{1}{N}\sum_{\alpha \in B850}^{N} C_{l1}(\rho^\alpha))$, excitons more delocalized in the membrane environments compared to detergent, as expected due to the higher level of static disorder and weaker electronic couplings in the B800 ring in detergent. A similar comparison for the B850 states shows a larger average delocalization of states in POPC membrane than in DOPC membrane and detergent. This seems to arise primarily from increased delocalization of the high-energy dark states of the B850 ring relative to the other environments.

Fig. 4 compares C_{l1} with increasing static disorder for three low-energy and three high-energy exciton states of the B850 ring in POPC membrane and detergent. We find that the order of the exciton delocalization changes depending on the level of static disorder. Among the high energy levels in POPC membrane, the k $= -6$ level is more delocalized than the k $= +5$ level at static disorder below 200 cm $^{-1}$ (Fig. 4 *b*). Above 200 cm $^{-1}$, this is reversed and the k $= +5$ level is more delocalized. In POPC membrane, there is a reduction in delocalization of some states in the low-energy manifold (k $= 0$ to k $= +4$) relative to the detergent states, as expected due to static disorder being greater in the B850 ring of the membrane. Some states in the high-energy manifold (k $= -5$ to k $= 9$) display increased delocalization in POPC membrane, a result of stronger electronic couplings in the B850 ring, which results in the high- and low-energy manifolds having a more comparable delocalization than in detergent. To quantify this, The difference between the average C_{l1} of the high-energy manifold and low-energy manifold is 4 for both membrane models and 6 for the detergent model. Thus, in detergent LH2, there is a clear distinction in the delocalization between the lower-energy exciton manifold and the high-energy manifold, which is less pronounced in membrane environments.

These calculations suggest that the membrane tends to preserve the symmetry of the excitonic structure of the B850 ring by tuning the delocalization of the high- and low-energy exciton manifolds, thereby enhancing a quantum feature of the system. Since an excitonic description is required to accurately predict energy-transfer rates in LH2, changes in exciton delocalization could manifest as changes in the energy-transfer pathways of an excitation (58), altering coherence dynamics in LH2 when embedded in the membrane. As the system evolves in time, exciton delocalization can change due to environmental interactions (59). More sophisticated measures can help verify whether these differences in delocalization from detergent to membrane persist over the inter-ring energy-transfer timescales.

Theoretical linear spectra

One of the key differences seen in experiments comparing detergent-isolated and membrane-embedded LH2 is the redshift of the B850 band in the linear absorption spectra of the LH2 (15–17). Fig. 5 *a* shows the B850 absorption and B800 fluorescence for membrane and detergent LH2 calculated using the same lineshape theory that is used to compute energy-transfer rates in GFT, and Fig. 5 *b* shows the same spectra calculated using HEOM.

The HEOM spectrum predicts the redshift of the B850 absorption peak for both POPC and DOPC membrane relative to detergent. Lineshape theory predicts the redshift for the POPC membrane; however, it predicts a blueshift for DOPC membrane relative to detergent. Comparing this with the exact results of the HEOM suggests that lineshape theory is not accurate enough to account for the subtle differences between the three environments. Conversely, HEOM is able to resolve the sensitive spectral differences between the environments.

Isolated BChl a absorbs at 800 nm, but when they come together to form the B850 ring, interchromophore electronic interactions shift the 800 nm absorption peak to 850 nm (1). Therefore, the observed shift in the B850 absorption spectrum between POPC membrane and detergent likely arises due to stronger interchromophore couplings and consequently increased delocalization of excitons in the membrane. In DOPC, the reduced redshift compared to POPC is possibly related to the intra-dimer B850 couplings being weaker than in detergent, whereas inter-dimer couplings are stronger.

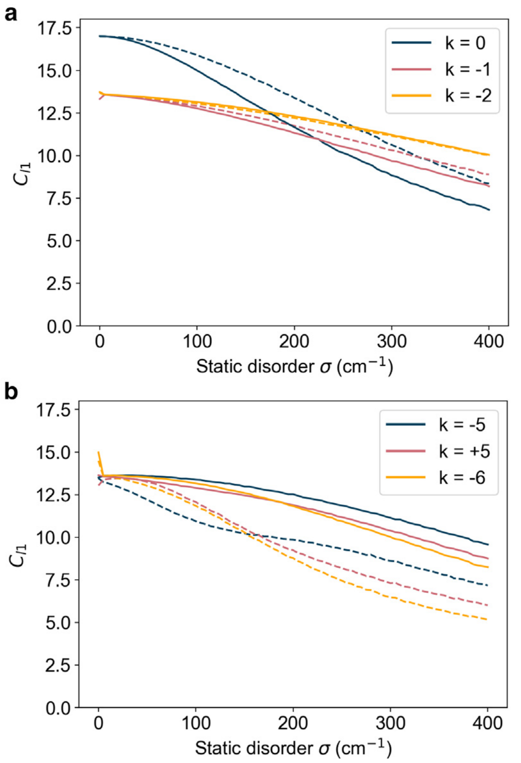

FIGURE 4 Exciton delocalisation vs static disorder. The average $l1$ norm of coherence of three levels selected from the (*a*) lower-energy exciton manifold and (*b*) high-energy exciton manifold of the B850 ring as a function of static disorder. Solid lines are for membrane-embedded LH2, and dotted lines are for the detergent-isolated LH2. The low-energy excitons show a reduced delocalization in the membrane, and the high-energy excitons have increased delocalized in the membrane. An average of over 10,000 realizations of static disorder was used to compute C_{l1}.

The redshift of the B850 band reduces the spectral overlap of the B800 fluorescence and B850 absorption bands, which would imply slower B800 to B850 energy-transfer times in the membrane. Since measured energy-transfer rates are faster in the membrane, this indicates that the increased delocalization of the B850 excitons compensates for the slightly reduced overlap. Of all the B850 excitons, the dark states show the greatest increase in delocalization in the membrane, hence playing an important role in the energy transfer.

B800 to B850 transfer rate distribution

The B800 to B850 energy-transfer rate was calculated for membrane-embedded LH2 and detergent-isolated LH2 using GFT (Eq. 22). 10,000 realizations of static disorder were used for each environment, and the distribution of the transfer rate over these realizations are shown in Fig. 6 *a*. In qualitative agreement with experimental work (15), the B800 to B850 transfer rate in POPC membrane LH2 has a faster average of 1.34 ps^{-1} (746 fs) compared to the rate in detergent where the average is 1.08 ps^{-1} (925 fs).

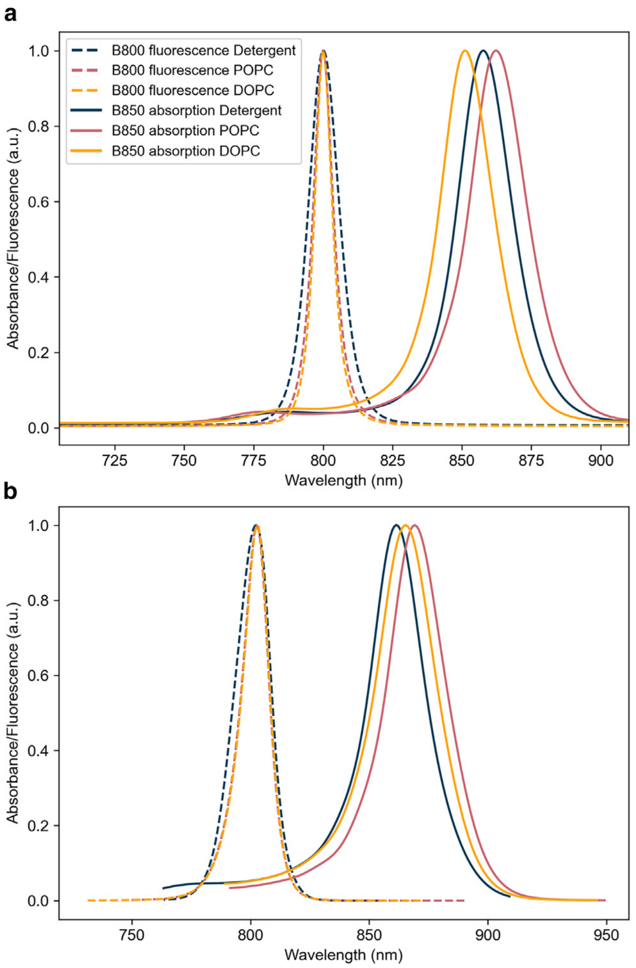

FIGURE 5 Theoretical absorption and fluorescence spectra. Theoretical linear spectra of the individual B800 and B850 rings computed using (*a*) lineshape theory as outlined in the GFT section, averaged over 2000 realizations of disorder, and (*b*) using HEOM as outlined in the Hierarchical equations of motion section, averaged over 1000 realizations of disorder. Convergence of the HEOM spectra is obtained when the hierarchy is truncated at tier 3 for B850 absorption and tier 4 for B800 fluorescence.

To corroborate rates obtained using GFT, estimates of the B800 to B850 energy-transfer rates for 2000 realizations of disorder have been computed using HEOM per the procedure outlined in the Hierarchical equations of motion section and are shown as a histogram in Fig. 6 *b*. In agreement with GFT rates, average energy transfer is found to be faster in POPC membrane at 1.04 ps^{-1} (962 fs) compared to detergent at 0.83 ps^{-1} (1.2 ps). Average rates obtained using HEOM are faster than those determined by GFT, which is likely a result of mapping the B800 to B850 transfer process to a one-step process for the HEOM-derived rates. Meanwhile GFT considers multiple, simultaneous B800 to B850 exciton transfer processes. Despite this discrepancy, there is still qualitative agreement between both the exact and perturbative rates, indicating that GFT is able to capture differences between detergent and membrane LH2.

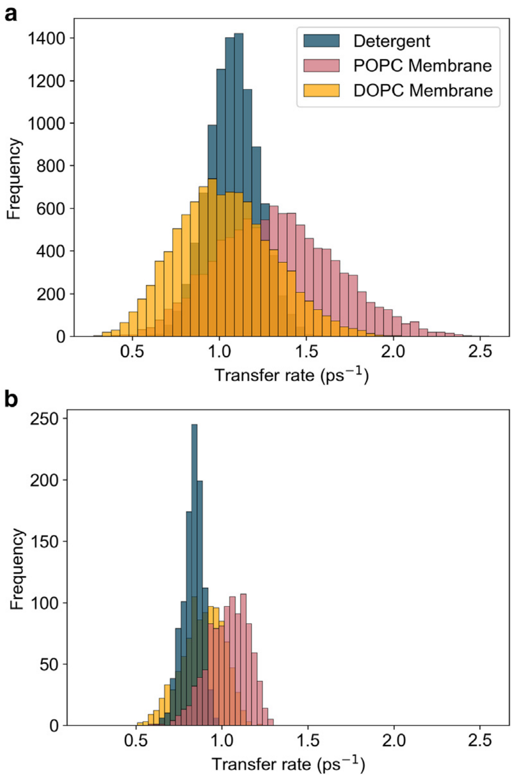

FIGURE 6 B800 to B850 energy transfer rate distribution. (*a*) Distribution of 10,000 realizations of the B800 to B850 energy-transfer rate calculated using GFT for detergent-isolated LH2 and two different lipid compositions of membrane-embedded LH2, DOPC, and POPC. Average transfer rates are 1.08 ps $^{-1}$, 1.04 ps $^{-1}$, and 1.34 ps $^{-1}$, respectively. Standard deviations are 0.14 ps $^{-1}$, 0.28 ps $^{-1}$, and 0.34 ps $^{-1}$, respectively. (*b*) Distribution of 2000 realizations of the B800 to B850 energy-transfer rate calculated using the fitting population equation Eq. 18 with rates obtained through HEOM. Average transfer rates are 0.83 ps $^{-1}$, 0.89 ps $^{-1}$, and 1.04 ps $^{-1}$, respectively. Standard deviations are 0.06 ps $^{-1}$, 0.12 ps $^{-1}$, and 0.12 ps $^{-1}$, respectively.

To understand the microscopic origin of the increased energy-transfer rate in membrane LH2, we computed the B800 to B850 energy-transfer rate for LH2 embedded in a membrane composed of a different lipid species, DOPC (36). In a DOPC membrane, the average B800 to B850 transfer rate is slower than in POPC membrane. This suggests that lipid species can influence energy-transfer rates within the LH2. There is evidence that changes in the lipid composition of the bilayer impact lateral pressure and electric field profiles, which leads to changes in the conformational equilibrium of membrane proteins (60,61). Phospholipids in the bacterial membrane that hosts the LH2 could present lateral pressure and electric field profiles that differ from the detergent environment, which may alter the electronic structure, and therefore function, of the LH2 (15).

GFT effectively distinguishes differences in the B800 to B850 energy transfer within the LH2 in different lipid compositions, making it a valuable tool for gaining qualitative insights into how the energy-transfer mechanisms vary in different environments. However, GFT has limitations when a drastic change in environment, such as from lipid to detergent, is considered. GFT predicts faster transfer rates in detergent than in DOPC membrane, contradicting experimental results that find faster transfer in a membrane environment. Meanwhile, HEOM-derived rates predict slower transfer in detergent, as expected from experimental observations. The discrepancy between the GFT and HEOM rates likely stems from two key approximations made in GFT that neglect contributions from nonequilibrium and coherent effects. Firstly, GFT assumes the initial state is limited to being localized on the B800 ring and that the excitation then "hops" to a state localized on the B850 ring. HEOM instead allows for the initial state to evolve coherently from the B800 ring to the B850 ring, thus allowing for a state to be delocalized over both rings during transfer. Secondly, GFT assumes the initial B800 state begins and remains in thermal equilibrium. In contrast, HEOM-derived rates account for nonequilibrium effects during transfer, as the interaction with the environment results in the initial state evolving and shifting out of equilibrium. HEOM-derived rates emphasize that nonequilibrium and coherent contributions to the B800 to B850 energy-transfer rate are key to accurately predicting differences in the LH2's function from detergent to membrane environments. However, GFT is able to consider multiple simultaneous excitation transfer processes from B800 to B850, whereas the procedure to obtain HEOM-derived rates maps B800 to B850 transfer onto the kinetics of a two-state system. Therefore, GFT remains a powerful theoretical framework for providing insights into the energy-transfer mechanisms within the LH2.

The broader distribution of the B800 to B850 energy-transfer rates for membrane LH2 indicates that, across an ensemble of LH2's, the transfer rate varies more in membrane than in detergent. The negative relationship between the energy-transfer rate and the standard deviation of the energy-transfer rate for LH2 in different environments suggests the possibility of a speed-accuracy trade-off within the LH2 (62). Trade-offs exist on a molecular level, with processes like protein synthesis prioritizing speed over fidelity (63). Such a trade-off is the result of the biological system possessing a trait that cannot increase without the decrease of another trait. For energy transfer in the LH2, the traits involved may be related to lipid properties determined by the lipid composition of the bacterial membrane. However, although a negative relationship is a prerequisite for a trade-off, it is not sufficient, and laboratory evolution experiments are required to identify whether a trade-off exists.

Dominating exciton transfer pathways

To understand the microscopic differences underlying the change in the B800 to B850 energy-transfer rate from detergent to membrane, the dominating exciton energy-transfer pathways were determined in each environment. We

identify the important transfer pathways as being between excitons with the fastest average exciton transfer rate, as they have the greatest influence on the average B800 to B850 transfer rate. The exciton transfer rate (Eq. 23) was determined between all combinations of donor B800 and acceptor B850 excitons and averaged over 10,000 realizations of disorder. For each realization, the exciton transfer rate is weighted by the thermal occupation of the donor state to correctly weight its contribution to the average B800 to B850 transfer rate (Eq. 22).

The dominant exciton energy-transfer pathways for B800 to B850 transfer in each environment are given in Table 4. In all environments, the dominant energy-transfer pathway is via dark B850 states, although the specific excitons are slightly different. This is due to differences in the spectral overlap of the excitons in each environment, which can be seen by the different relative positions of the B800 and B850 average energy levels in Fig. 3. In the POPC membrane, the B800 levels overlap with higher-energy B850 states than in detergent. As a result, the dominant energy-transfer pathways for B800 to B850 transfer are altered compared to detergent LH2. Despite the B800 to B850 transfer rate being slowest in DOPC membrane, the dominant pathway in DOPC membrane is faster than the pathway in detergent. We find that, in detergent, multiple pathways with moderate transfer rates (0.03 ps^{-1}) exist from low-energy B800 levels, whereas in DOPC membrane transfer from those levels is much slower (>0.01 ps^{-1}). Thus, GFT predicts the overall B800 to B850 rate to be faster in detergent than DOPC due to the increased number of available pathways for an excitation to take.

Previously, we showed that, on average, the delocalization of B850 excitons is greater in membrane LH2. The delocalization of the B850 excitons that are key energy acceptors in B800 to B850 energy transfer is of greater importance, as it allows us to assess whether the change in delocalization is relevant to the change in energy transfer that we see in membrane LH2. We find that, in the membrane, the important B850 excitons are on average more delocalized ($C_{l1} = 12$) than the equivalent in detergent ($C_{l1} = 11$). This suggests that coherent dynamics in LH2 may be altered when embedded in the membrane. Further investigation would require the use of HEOM, as GFT does not provide information on coherent dynamics.

The exciton transfer rate entering GFT depends on the exciton coupling strength squared and the spectral overlap between the exciton lineshapes. Looking at how these properties change from detergent to membrane can help identify which specific differences in membrane contribute to an increased average transfer rate and broader distribution. The distribution of 10,000 realizations of the exciton transfer rate, the exciton coupling, and the exciton spectral overlap was determined for the dominating pathway in each environment and is given in Fig. 7 *a–c* with average values listed in Table 4. For comparison, the same exciton properties are given in Fig. 7 *d–f* for states D = 1 to k = −2, a pair of states that have a slow exciton transfer rate and are therefore considered nondominating. Average values for the nondominating excitons are given in Table 5.

For the dominating excitons, although the average spectral overlap is comparable in all three environments, the average exciton coupling is strongest in POPC membrane. The exciton transfer rate scales with the exciton coupling, such that the fastest transfer rate is between the POPC membrane donor and acceptor pair, indicating that the dominant energy-transfer pathway is mostly dependent on the exciton coupling strength. The exciton coupling strength scales with the electronic coupling between nearest-neighbor B800 and B850 chromophore sites (Table 1), suggesting that stronger interchromophore electronic coupling is the main factor contributing to the faster energy-transfer rate in membrane. Stronger electronic coupling between B800 and B850 chromophore transition dipole moments could arise from a change in the direction of the dipoles or a reduced distance between them. By comparing the position coordinates of the B850 chromophores in each model, neighboring B850 chromophores are slightly closer in the membrane LH2 model, such that stronger interchromophore electronic couplings would be expected.

The exciton coupling is additionally dependent on the exciton delocalization scaled by the electronic coupling (Eq. 24). Increased delocalization of the excitons can contribute to the stronger interaction between excitons by spreading an excitation over a greater number of electronically interacting sites; however, the strength of the interaction between those sites is also important. Although the acceptor B850 exciton is delocalized similarly in DOPC membrane and in detergent, the donor B800 exciton is more delocalized in DOPC. This suggests that the B800 exciton delocalization is still relevant for energy transfer despite being smaller than the B850 exciton delocalization.

Although the form of the distribution of the exciton coupling is similar in each environment, the distribution

TABLE 4 The B800-B850 exciton pair that provides the fastest energy-transfer pathway from B800 to B850 in each environment, their exciton transfer rate, exciton coupling, spectral overlap, and the *l*1 norm of coherence of the donor and acceptor states averaged over 10,000 realizations of disorder

LH2 Environment	B800 State	B850 State	$P_\alpha k_{\alpha\beta}$ (ps^{-1})	$k_{\alpha\beta}$ (ps^{-1})	$\lvert V_{\alpha\beta}\rvert^2$ (cm^{-1})	$\Theta_{\alpha\beta}$	C_{l1}^{B850}	C_{l1}^{B800}
Membrane POPC	D = 7	k = +5	0.11	1.39	66	0.016	11	5
Membrane DOPC	D = 7	k = +5	0.06	0.77	63	0.013	10	5
Detergent	D = 7	k = −5	0.04	0.43	56	0.013	10	2

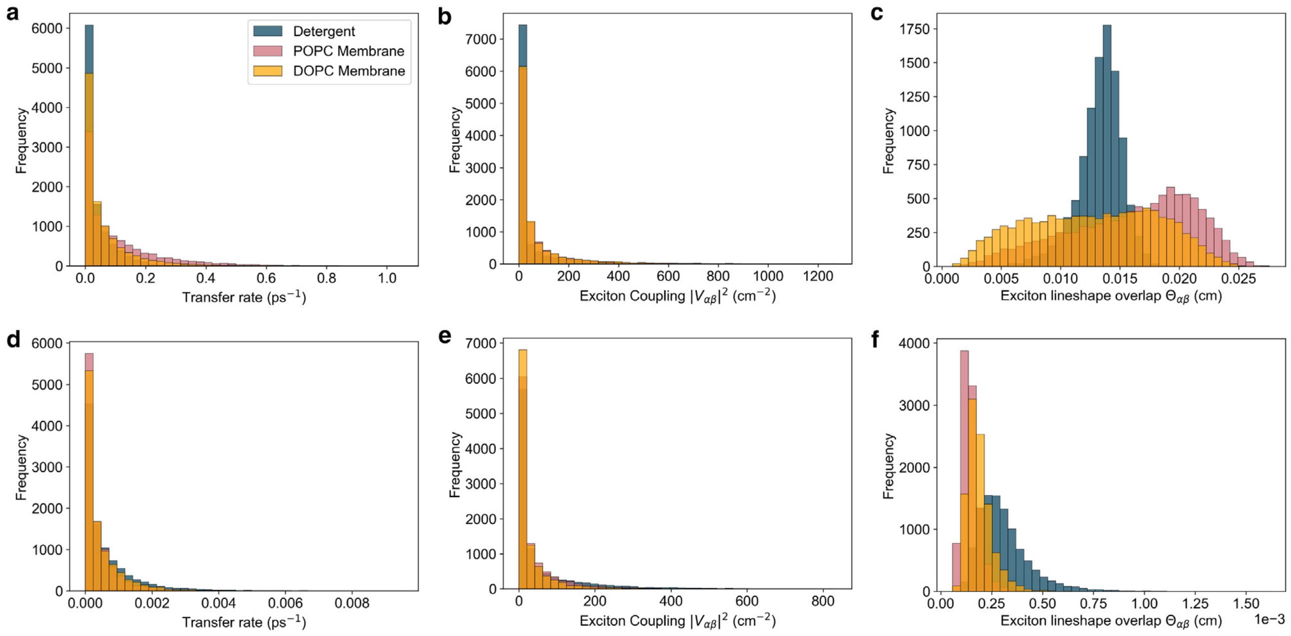

FIGURE 7 Distribution of key exciton properties. Exciton properties for (*a–c*) exciton pairs that dominate the B800 to B850 energy transfer and (*d–f*) exciton pairs that have a small contribution to the B800 to B850 energy-transfer rate (nondominating). Distribution of 10,000 realizations of the exciton energy-transfer rate, the exciton coupling, and the exciton spectral overlap between a B800 exciton to a B850 exciton. Transfer rates are calculated using Eq. 23 and weighted by the thermal occupation of the donor B800 state. The dominating excitons are D = 7 to k = +5 for POPC and DOPC membrane LH2 and D = 7 to k = −5 for detergent LH2. The nondominating excitons are D = 1 to k = −2.

of the spectral overlap (Fig. 7 *c* is where differences emerge. The spectral overlap distribution peaks sharply for the detergent but is broad and flat for POPC membrane and DOPC membrane, which contributes to a greater variation in the exciton transfer rate in the membrane. At first, it may seem that this is a consequence of higher static disorder in the membrane, which, through random shifts in the relative positions of the donor and acceptor energy levels, would result in a greater variation of the energy gap between donor and acceptor. However, the distribution of the donor-acceptor energy gap $\omega_{\alpha\beta}$ is similar in all three environments (Fig. S1). Additionally, the B800 to B850 transfer rate computed using the same level of static disorder in each ring for detergent and membrane environments still produces a broader distribution and faster average rate for the POPC membrane, suggesting that higher levels of static disorder in the membrane are unlikely to be the cause of the broadened distribution (Fig. S2). The exciton spectral overlap also depends on the exciton environmental parameters $\lambda_{\alpha\alpha\alpha\alpha}$ and $g_{\alpha\alpha\alpha\alpha}(t)$, which are determined by exciton delocalization scaled by the site environmental parameters λ_i and g_i via $\lambda_{\alpha\alpha\alpha\alpha} \propto \lambda_i/\mathrm{IPR}$. We have computed the B800 to B850 energy-transfer rate using the same environmental parameters for both membrane and detergent models and found that a broader distribution and faster rate in membrane LH2 still holds (Fig. S3). This points to the change in electronic properties being of high importance to the changes in energy transfer in membrane LH2.

For the dominant exciton transfer pathways, we see that the excitonic coupling is the key factor determining the exciton energy-transfer rate. The excitonic coupling is dependent on both the electronic coupling between sites in the B800 ring to sites in the B850 ring and the delocalization of the B800 and B850 exciton in question. A figure of merit that captures both these electronic properties is given by the inter-ring electronic coupling scaled by the geometric average delocalization of the important B800 and B850 exciton pair, $\sqrt{\overline{C_{l1}^{\mathrm{B800}}}\,\overline{C_{l1}^{\mathrm{B850}}}} V_{B800,\alpha_2}$. Although V_{B800,α_2} provides information on the interactions between the rings,

TABLE 5 A B800-B850 exciton pair that has slow exciton energy transfer from B800 to B850, their exciton transfer rate, exciton coupling, spectral overlap and the *l*1 norm of coherence of the donor and acceptor states averaged over 10,000 realizations of disorder

LH2 Environment	B800 State	B850 State	$P_\alpha k_{\alpha\beta}$ (ps^{-1})	$k_{\alpha\beta}$ (ps^{-1})	$\lvert V_{\alpha\beta}\rvert^2$ (cm^{-1})	$\Theta_{\alpha\beta}$	C_{l1}^{B850}	C_{l1}^{B800}
Membrane POPC	D = 1	k = −2	0.00035	0.0024	38	1.4	12	4
Membrane DOPC	D = 1	k = −2	0.00043	0.0031	28	1.9	11	4
Detergent	D = 1	k = −2	0.0006	0.0043	57	3.1	12	3

$\sqrt{\overline{C_{l1}^{B800}}\ \overline{C_{l1}^{B850}}}$ is a result of the site energies and electronic couplings within each ring. Table 6 lists this figure of merit for the dominating pathways in each environment, using both the IPR and C_{l1} as a measure of delocalization.

The increase in the figure of merit correlates with the increase in exciton transfer rate of the dominant exciton pairs from detergent to POPC membrane. It allows us to relate the change in transfer rate directly to the B800 to B850 interchromophore electronic couplings and delocalization. For POPC membrane, although V_{B800,α_2} is largest, the geometric average IPR of the B800 and B850 exciton pairs suggests that an increased delocalization of the B850 and B800 excitons in POPC membrane also contributes to an increased exciton transfer rate.

In the case of the nondominant excitons, although the exciton coupling is stronger in POPC than DOPC membrane, the transfer rate is faster in DOPC. There is instead a correlation between the exciton transfer rate and the spectral overlap between the exciton lineshape functions. The spectral overlap is dependent on the energy gap between the excitons and the width of the lineshape, which is determined by the real part of $g_\alpha(t)$. $g_\alpha(t)$ is given by $g_i(t)$ scaled by $1/\mathrm{IPR}$. In detergent, the B800 exciton is highly localized with an IPR of 3 resulting in a broader lineshape. Additionally, the energy gap between the donor and acceptor exciton is smallest in detergent, resulting in a greater spectral overlap. Thus, we see that, for the nondominant pathways, the spectral overlap is the dominating factor in determining the exciton transfer rate.

These results suggest that, although there is an interplay between the exciton coupling strength and spectral overlap when determining the exciton transfer rate, the dominating transfer pathways in B800 to B850 transfer depend strongly on exciton coupling strength alone. Thus, the real interplay is between the B800 to B850 inter-ring coupling and the delocalization of excitons in each ring, two properties that can be traced back to the electronic properties of the LH2. Thus, changes in the electronic properties from detergent to membrane environments alter exciton energy-transfer pathways, impacting the overall B800 to B850 transfer rate.

DISCUSSION

So far, knowledge of the structure and function of the LH2 complex has been gained mostly through investigating complexes isolated from their native environment in the photosynthetic membrane. Recent experimental studies have found that energy transfer within the complex is faster in a membrane environment that mimics the bacterial membrane (15). Using two levels of theory, GFT and HEOM, to estimate B800 to B850 energy-transfer rates, we have demonstrated how faster energy transfer in membrane-embedded LH2 can be linked to changes in the electronic properties of the complex. In agreement with previous theoretical studies, we have identified that the dominating pathway an excitation takes from the B800 to the B850 ring is via the dark B850* states, and we find this to be the case in both membrane and detergent environments (7). We have shown how faster energy transfer in the membrane is the result of the increased delocalization and stronger coupling between the excitons involved in the dominating pathways. Signatures of stronger electronic coupling in the B850 ring are additionally present in both experimental and theoretical linear spectra, which show a red shift in the B850 absorption, a change characteristic of stronger interchromophore electronic couplings (15–17). Finally, we find a broader distribution of the B800 to B850 energy-transfer rates for an ensemble of 10,000 LH2s and suggest that a biological trade-off may be present that allows the LH2 to achieve faster average energy transfer in membrane through a broad spread of energy-transfer rates.

We use both GFT and HEOM to determine the average B800 to B850 energy-transfer rate in detergent and POPC membrane environments and find a qualitative agreement between the two approaches indicating a faster transfer rate in membrane LH2, in agreement with experimental pump-probe measurements (15). The authors suggest that lipid bilayer properties such as the lateral pressure profile of the membrane or a hydrophobic (mis)match may be the microscopic origin of the increased energy-transfer rate in the membrane. The membrane lipid bilayer provides stability to the LH2 complex through lateral pressure, which is altered when in detergent or in varying membrane lipid compositions (61,64). To assess the importance of lipid-protein interactions on the energy transfer within the LH2, we computed the average B800 to B850 transfer rate for LH2 embedded in two different lipids, POPC and DOPC. Both GFT and HEOM rates predict slower transfer in DOPC compared to POPC, indicating that changes in the lipid

TABLE 6 Figure of merit that captures the electronic properties of the LH2 through the electronic coupling between the B800 and B850 ring and the delocalization of excitons within each ring

LH2 Environment	$\sqrt{\overline{C_{l1}^{B800}}\ \overline{C_{l1}^{B850}}}V_{B800,\alpha_2}$	$\sqrt{\overline{IPR^{B800}}\ \overline{IPR^{B850}}}V_{B800,\alpha_2}$	$\sqrt{\overline{C_{l1}^{B800}}\ \overline{C_{l1}^{B850}}}$	$\sqrt{\overline{IPR^{B800}}\ \overline{IPR^{B850}}}$
Membrane POPC	285	215	6.8	6
Membrane DOPC	264	191	6.9	5
Detergent	152	135	4.8	4

The figure of merit for the dominating exciton transfer pathways in each environment. The increase in the transfer rate from detergent to membrane correlates with the increase in the figure of merit, as expected, since changes in transfer rate between each environment are strongly dependent on changes in the electronic properties of the LH2.

composition can result in changes in energy transfer. Understanding how the energy transfer within the LH2 changes as a function of the lipid properties could reveal how the complex achieves faster rates of energy transfer in a lipid environment. Although energy transfer is expected to be slowest in detergent as predicted by the HEOM-derived rate, GFT predicts transfer to be slowest in DOPC. This discrepancy highlights the importance of nonperturbative frameworks to resolve and understand the differences of energy-transfer kinetics and to rationalize experimental observations. We note that the estimated transfer times with HEOM are in general longer than with GFT. These quantitative differences result in part from the fact that, in the HEOM approach, we map the B800 to B850 transfer process onto the kinetics of a two-state system. Rigorous approaches to extracting more accurate transfer rates from a nonperturbative framework such as HEOM is an open problem (65) that goes beyond the scope of the current manuscript and will be presented elsewhere.

An advantage of GFT is that the underlying excitonic properties can be studied to pinpoint changes that could result in faster B800 to B850 energy transfer. The dominating transfer pathways in the LH2 indicate that faster exciton transfer in the membrane can be linked to stronger excitonic couplings, a direct result of both stronger interchromophore electronic couplings and increased delocalization of excitations. The membrane lipid bilayer provides stability to the LH2 complex through lateral pressure, which is altered when in detergent. Differences in lipid-protein and detergent-protein interactions could alter electronic couplings via perturbations in the geometry of the chromophores, as the protein scaffold has control over the position and orientation of the chromophores. Methods more sophisticated than the dipole-dipole approximation are used to determine all the electronic couplings in the membrane LH2 models that account for screening due to the lipid environment, such that a closer packing of B850 chromophores may not be the sole reason for stronger electronic couplings (26,36). Accompanying stronger interchromophore electronic couplings is the increased delocalization of the excitons dominating B800 to B850 energy transfer in membrane. Two-dimensional spectroscopy measurements have found quantum beating in the fluorescence signals of the LH2 from *R. acidophilus*, a signature of quantum coherent dynamics (66). Quantum-beating signals arise as a result of constructive and destructive interference between different donor to single acceptor pathways over time. Such interference becomes possible when excitons are highly delocalized such that many relaxation pathways are available. Theoretical studies of model exciton systems suggest that interference is important to achieve high energy-trapping efficiency (67). Interference becomes possible when excitons are highly delocalized such that many relaxation pathways are available. Thus, the increased delocalization of excitons in membrane LH2 could impact the coherent dynamics within the complex.

The dark B850* states seem to be an important energy acceptor for B800 to B850 energy transfer within the LH2 in both detergent and membrane environments. Previous theoretical studies have found that B800 to B850* energy transfer occurs faster (600–800 fs) than transfer to lower-energy bright B850 exciton states (1 ps) (7). Energy-transfer pathways in the LH2 have been probed experimentally using two-dimensional spectroscopy, but it is difficult to detect a B800 to B850* signal since the third-order nonlinear response measured is proportional to the fourth power of the transition dipole moment, which, in the case of B850*, is negligible (8). Fidler et al. suggests that the excitons involved in this energy-transfer pathway may have parallel transition dipole moments, which would also prevent their detection. Despite its elusiveness, the presence of a fast B800 to B850* energy-transfer pathway could explain the additional fast-decay channel found when exciting LH2 at the blue end of the B800 band in hole-burning experiments (68–70). A similar pathway has been proposed in the LH3 complex from *R. acidophilus* strain 7050, a low-light variant of *R. acidophilus* (28). The LH3 has a similar nonameric structure to the LH2 but with the B850 band blue shifted to 820 nm, indicating that this mechanism may be shared across different variants of the complex. Studies on artificial light-harvesting systems have shown that transfer of excitation energy from bright to dark states may be used to prevent re-emission since the dark state cannot optically decay, thus increasing the efficiency of energy transfer in the system (71–73). The dark B850* states may play a similar role in trapping absorbed solar energy by quickly moving excitation energy out of the B800 ring, where it would otherwise relax to low-energy B800 states that have a greater transition dipole strength.

By calculating the B800 to B850 energy-transfer rate for several thousands of realizations of static disorder, we also resolve the heterogeneity across an ensemble of LH2 complexes and can study the form of their distribution. We find a broader distribution of energy-transfer rates in membrane-embedded LH2, suggesting that the energy-transfer mechanism has a lower level of precision in the native environment due to the greater standard deviation of the transfer rates compared to detergent. A concept used to understand the relationship between different traits in a biological system is a trade-off, which can be identified by a negative relationship between two traits (62). The distribution of the transfer rate in membrane and detergent LH2 implies that the complex sacrifices precision in the transfer rate for speed. The traits underlying such a trade-off would likely be related to properties of the LH2 that change from detergent to membrane. However, identifying a trade-off would require more thorough investigation and laboratory evolution experiments.

In summary, we have shown that increased energy-transfer efficiency within membrane-embedded LH2 can be traced back to altered energy-transfer pathways and enhanced

quantum delocalization of excitations within the complex. Further work toward understanding the biological interactions underlying such enhancements will not only provide a deeper understanding of the function of the LH2 but will also lead to improved theoretical tools to study similar photosynthetic complexes. Our choice of parameters has been justified on the basis of experiment-theory consistency. Currently, there is a lack of electronic parameters for LH2 in detergent and in membrane, which are derived on the same quantum chemical framework; this is because there is a notable gap in the field when it comes to force fields specifically designed for LH2 in detergent, and their development is a complex and nontrivial task beyond the scope of paper. Our study motivates further research in this direction. Additionally, to study the LH2 in its biological environment, having a nonperturbative framework that can yield excitation transfer rates is essential. We believe the work presented here is a first step toward addressing these challenges and uncovering the role that the biological environment plays in the efficiency of these light-harvesting complexes.

DATA AND CODE AVAILABILITY

The Python code developed during this study and further data are available from the corresponding authors upon reasonable request.

ACKNOWLEDGMENTS

We thank Gabriela Schlau-Cohen and Charlie Nation for insightful discussions. We gratefully acknowledge financial support from the Engineering and Physical Sciences Research Council (EPSRC UK) grant EP/T517793/1 and from the Gordon and Betty Moore Foundation grant 8820. The authors acknowledge use of the UCL Myriad High Performance Computing Facility.

AUTHOR CONTRIBUTIONS

A.O.-C. designed and supervised the research. C.K. and H.Ó.G. carried out the simulations. L.C. and B.M. provided quantum chemical insight. C.K. wrote the first draft. All authors analyzed the data, discussed the results, and contributed to the final version of the manuscript.

DECLARATION OF INTERESTS

The authors declare no competing interests.

SUPPORTING MATERIAL

Supporting material can be found online at https://doi.org/10.1016/j.bpj.2025.01.011.

REFERENCES

1. Cogdell, R. J., A. Gall, and J. Köhler. 2006. The architecture and function of the light-harvesting apparatus of purple bacteria: from single molecules to in vivo membranes. *Q. Rev. Biophys.* 39:227–324. https://doi.org/10.1017/S0033583506004434.
2. Sturgis, J. N., and R. A. Niederman. 2008. Atomic force microscopy reveals multiple patterns of antenna organization in purple bacteria: implications for energy transduction mechanisms and membrane modeling. *Photosynth. Res.* 95:269–278. https://doi.org/10.1007/s11120-007-9239-0.
3. Scheuring, S., J.-L. Rigaud, and J. N. Sturgis. 2004. Variable LH2 stoichiometry and core clustering in native membranes of Rhodospirillum photometricum. *EMBO J.* 23:4127–4133. https://doi.org/10.1038/sj.emboj.7600429.
4. Bahatyrova, S., R. N. Frese, ..., C. N. Hunter. 2004. The native architecture of a photosynthetic membrane. *Nature.* 430:1058–1062. https://doi.org/10.1038/nature02823.
5. Papiz, M. Z., S. M. Prince, ..., N. W. Isaacs. 2003. The structure and thermal motion of the B800–850 LH2 complex from Rps. acidophila at 2.0 Å resolution and 100 K: new structural features and functionally relevant motions. *J. Mol. Biol.* 326:1523–1538. https://doi.org/10.1016/S0022-2836(03)00024-X.
6. McDermott, G., S. M. Prince, ..., N. W. Isaacs. 1995. Crystal structure of an integral membrane light-harvesting complex from photosynthetic bacteria. *Nature.* 374:517–521. https://doi.org/10.1038/374517a0.
7. Novoderezhkin, V., M. Wendling, and R. Van Grondelle. 2003. Intra- and Interband Transfers in the B800- B850 Antenna of Rhodospirillum m olischianum: Redfield Theory Modeling of Polarized Pump- Probe Kinetics. *J. Phys. Chem. B.* 107:11534–11548. https://doi.org/10.1021/jp035432l.
8. Fidler, A. F., V. P. Singh, ..., G. S. Engel. 2013. Probing energy transfer events in the light harvesting complex 2 (LH2) of Rhodobacter sphaeroides with two-dimensional spectroscopy. *J. Chem. Phys.* 139:155101. https://doi.org/10.1063/1.4824637.
9. Massey, S. C., P.-C. Ting, ..., G. S. Engel. 2019. Orientational dynamics of transition dipoles and exciton relaxation in LH2 from ultrafast two-dimensional anisotropy. *J. Phys. Chem. Lett.* 10:270–277. https://doi.org/10.1021/acs.jpclett.8b03223.
10. Sundström, V., T. Pullerits, and R. van Grondelle. 1999. Photosynthetic light-harvesting: reconciling dynamics and structure of purple bacterial LH2 reveals function of photosynthetic unit. *J. Phys. Chem. B.* 103:2327–2346. https://doi.org/10.1021/jp983722+.
11. Georgakopoulou, S., R. N. Frese, ..., G. van der Zwan. 2002. Absorption and CD spectroscopy and modeling of various LH2 complexes from purple bacteria. *Biophys. J.* 82:2184–2197. https://doi.org/10.1016/S0006-3495(02)75565-3.
12. Jimenez, R., S. N. Dikshit, ..., G. R. Fleming. 1996. Electronic excitation transfer in the LH2 complex of Rhodobacter sphaeroides. *J. Phys. Chem.* 100:6825–6834. https://doi.org/10.1021/jp953074j.
13. Alden, R. G., E. Johnson, ..., R. G. Cogdell. 1997. Calculations of Spectroscopic Properties of the LH2 Bacteriochlorophyll- Protein Antenna Complex from Rhodopseudomonas Acidophila. *J. Phys. Chem. B.* 101:4667–4680. https://doi.org/10.1021/jp970005r.
14. Wu, H.-M., M. Ratsep, ..., G. J. Small. 1997. Comparison of the LH2 antenna complexes of Rhodopseudomonas acidophila (strain 10050) and Rhodobacter sphaeroides by high-pressure absorption, high-pressure hole burning, and temperature-dependent absorption spectroscopies. *J. Phys. Chem. B.* 101:7641–7653. https://doi.org/10.1021/jp9715134.
15. Ogren, J. I., A. L. Tong, ..., G. S. Schlau-Cohen. 2018. Impact of the lipid bilayer on energy transfer kinetics in the photosynthetic protein LH2. *Chem. Sci.* 9:3095–3104. https://doi.org/10.1039/C7SC04814A.
16. Freiberg, A., M. Rätsep, and K. Timpmann. 2012. A comparative spectroscopic and kinetic study of photoexcitations in detergent-isolated and membrane-embedded LH2 light-harvesting complexes. *Biochim. Biophys. Acta Bioenerg.* 1817:1471–1482. https://doi.org/10.1016/j.bbabio.2011.11.019.
17. Agarwal, R., A. H. Rizvi, ..., G. R. Fleming. 2002. Nature of disorder and inter-complex energy transfer in LH2 at room temperature: a three pulse photon echo peak shift study. *J. Phys. Chem. A.* 106:7573–7578. https://doi.org/10.1021/jp014054m.

18. Richter, M. F., J. Baier, …, S. Oellerich. 2007. Single-molecule spectroscopic characterization of light-harvesting 2 complexes reconstituted into model membranes. *Biophys. J.* 93:183–191. https://doi.org/10.1529/biophysj.106.103606.

19. Hunter, C. N., F. Daldal, …, J. T. Beatty. 2009. The Purple Phototrophic Bacteria, volume 28. Springer, Dordrecht. https://doi.org/10.1007/978-1-4020-8815-5.

20. Nagatsuma, S., K. Gotou, …, Z.-Y. Wang-Otomo. 2019. Phospholipid distributions in purple phototrophic bacteria and LH1-RC core complexes. *BBA Bioenergetics.* 1860:461–468. https://doi.org/10.1016/j.bbabio.2019.04.001.

21. Dewa, T., A. Sumino, …, M. Nango. 2013. Energy transfer and clustering of photosynthetic light-harvesting complexes in reconstituted lipid membranes. *Chem. Phys.* 419:200–204. https://doi.org/10.1016/j.chemphys.2012.12.039.

22. Sumino, A., T. Dewa, …, M. Nango. 2013. Influence of phospholipid composition on self-assembly and energy-transfer efficiency in networks of light-harvesting 2 complexes. *J. Phys. Chem. B.* 117:10395–10404. https://doi.org/10.1021/jp4047819.

23. Rigaud, J.-L. 2002. Membrane proteins: functional and structural studies using reconstituted proteoliposomes and 2-D crystals. *Braz. J. Med. Biol. Res.* 35:753–766.

24. Pflock, T., M. Dezi, …, S. Oellerich. 2008. Comparison of the fluorescence kinetics of detergent-solubilized and membrane-reconstituted LH2 complexes from Rps. acidophila and Rb. sphaeroides. *Photosynth. Res.* 95:291–298. https://doi.org/10.1007/s11120-007-9245-2.

25. Pflock, T. J., S. Oellerich, …, J. Köhler. 2011. The electronically excited states of LH2 complexes from Rhodopseudomonas acidophila strain 10050 studied by time-resolved spectroscopy and dynamic Monte Carlo simulations. II. Homo-arrays of LH2 complexes reconstituted into phospholipid model membranes. *J. Phys. Chem. B.* 115:8821–8831. https://doi.org/10.1021/jp2023583.

26. Cupellini, L., S. Jurinovich, …, B. Mennucci. 2016. An ab initio description of the excitonic properties of LH2 and their temperature dependence. *J. Phys. Chem. B.* 120:11348–11359. https://doi.org/10.1021/acs.jpcb.6b06585.

27. Kennis, J. T. M., A. M. Streltsov, …, J. Amesz. 1997. Femtosecond dynamics in isolated LH2 complexes of various species of purple bacteria. *J. Phys. Chem. B.* 101:7827–7834. https://doi.org/10.1021/jp963359b.

28. Tong, A. L., O. C. Fiebig, …, G. S. Schlau-Cohen. 2020. Comparison of the energy-transfer rates in structural and spectral variants of the B800–850 complex from purple bacteria. *J. Phys. Chem. B.* 124:1460–1469. https://doi.org/10.1021/acs.jpcb.9b11899.

29. Freiberg, A., M. Rätsep, …, G. Trinkunas. 2009. Excitonic polarons in quasi-one-dimensional LH1 and LH2 bacteriochlorophyll a antenna aggregates from photosynthetic bacteria: A wavelength-dependent selective spectroscopy study. *Chem. Phys.* 357:102–112. https://doi.org/10.1016/j.chemphys.2008.10.043.

30. Scholes, G. D., X. J. Jordanides, and G. R. Fleming. 2001. Adapting the Förster theory of energy transfer for modeling dynamics in aggregated molecular assemblies. *J. Phys. Chem. B.* 105:1640–1651. https://doi.org/10.1021/jp003571m.

31. Davydov, A. S. 1964. The theory of molecular excitons. *Sov. Phys. Usp.* 7:145–178. https://doi.org/10.1007/978-1-4899-5169-4.

32. Valkunas, L., D. Abramavicius, and T. Mančal. 2013. Microscopic Theory of Linear Absorption and Fluorescence, 14. John Wiley & Sons, Ltd, Weinheim, pp. 295–314, chapter. https://onlinelibrary.wiley.com/doi/abs/10.1002/9783527653652.ch14.

33. Krueger, B. P., G. D. Scholes, and G. R. Fleming. 1998. Calculation of couplings and energy-transfer pathways between the pigments of LH2 by the ab initio transition density cube method. *J. Phys. Chem. B.* 102:5378–5386. https://doi.org/10.1021/jp9811171.

34. Tretiak, S., C. Middleton, …, S. Mukamel. 2000. Bacteriochlorophyll and carotenoid excitonic couplings in the LH2 system of purple bacteria. *J. Phys. Chem. B.* 104:9540–9553. https://doi.org/10.1021/jp001585m.

35. Curutchet, C., J. Kongsted, …, B. Mennucci. 2011. Photosynthetic light-harvesting is tuned by the heterogeneous polarizable environment of the protein. *J. Am. Chem. Soc.* 133:3078–3084. https://doi.org/10.1021/ja110053y.

36. Cardoso Ramos, F., M. Nottoli, …, B. Mennucci. 2019. The molecular mechanisms of light adaption in light-harvesting complexes of purple bacteria revealed by a multiscale modeling. *Chem. Sci.* 10:9650–9662. https://doi.org/10.1039/C9SC02886B.

37. Sauer, K., R. J. Cogdell, …, H. Scheer. 1996. Structure-based calculations of the optical spectra of the LH2 bacteriochlorophyll-protein complex from Rhodopseudomonas acidophila. *Photochem. Photobiol.* 64:564–576. https://doi.org/10.1111/j.1751-1097.1996.tb03106.x.

38. May, V., and O. Kühn. 2008. Charge and Energy Transfer Dynamics in Molecular Systems. John Wiley & Sons, Weinheim. https://doi.org/10.1002/9783527602575.

39. Jang, S., S. E. Dempster, and R. J. Silbey. 2001. Characterization of the static disorder in the B850 band of LH2. *J. Phys. Chem. B.* 105:6655–6665. https://doi.org/10.1021/jp010169e.

40. Jang, S., R. J. Silbey, …, J. Köhler. 2011. Is there elliptic distortion in the light harvesting complex 2 of purple bacteria? *J. Phys. Chem. B.* 115:12947–12953. https://doi.org/10.1021/jp202344s.

41. Hofmann, C., T. J. Aartsma, and J. Köhler. 2004. Energetic disorder and the B850-exciton states of individual light-harvesting 2 complexes from Rhodopseudomonas acidophila. *Chem. Phys. Lett.* 395:373–378. https://doi.org/10.1016/j.cplett.2004.08.020.

42. van Oijen, A. M., M. Ketelaars, …, J. Schmidt. 1999. Unraveling the electronic structure of individual photosynthetic pigment-protein complexes. *Science.* 285:400–402. https://doi.org/10.1126/science.285.5426.400.

43. Novoderezhkin, V. I., D. Rutkauskas, and R. van Grondelle. 2006. Dynamics of the emission spectrum of a single LH2 complex: interplay of slow and fast nuclear motions. *Biophys. J.* 90:2890–2902. https://doi.org/10.1529/biophysj.105.072652.

44. Novoderezhkin, V., R. Monshouwer, and R. van Grondelle. 1999. Exciton (de) localization in the LH2 antenna of Rhodobacter sphaeroides as revealed by relative difference absorption measurements of the LH2 antenna and the B820 subunit. *J. Phys. Chem. B.* 103:10540–10548. https://doi.org/10.1021/jp9844415.

45. Monshouwer, R., M. Abrahamsson, R. van Grondelle…, 1997. Superradiance and exciton delocalization in bacterial photosynthetic light-harvesting systems. *J. Phys. Chem. B.* 101:7241–7248. https://doi.org/10.1021/jp963377t.

46. Baumgratz, T., M. Cramer, and M. B. Plenio. 2014. Quantifying coherence. *Phys. Rev. Lett.* 113:140401. https://doi.org/10.1103/PhysRevLett.113.140401.

47. Streltsov, A., G. Adesso, and M. B. Plenio. 2017. Colloquium: Quantum coherence as a resource. *Rev. Mod. Phys.* 89:041003. https://doi.org/10.1103/RevModPhys.89.041003.

48. Tanimura, Y., and R. Kubo. 1989. Time Evolution of a Quantum System in Contact with a Nearly Gaussian-Markoffian Noise Bath. *J. Phys. Soc. Jpn.* 58:101–114. https://doi.org/10.1143/JPSJ.58.101.

49. Tanimura, Y. 2020. Numerically "exact" approach to open quantum dynamics: The hierarchical equations of motion (HEOM). *J. Chem. Phys.* 153:020901. https://doi.org/10.1063/5.0011599.

50. Ishizaki, A., and Y. Tanimura. 2005. Quantum Dynamics of System Strongly Coupled to Low-Temperature Colored Noise Bath: Reduced Hierarchy Equations Approach. *J. Phys. Soc. Jpn.* 74:3131–3134. https://doi.org/10.1143/JPSJ.74.3131.

51. Shi, Q., L. Chen, …, Y. Yan. 2009. Efficient hierarchical Liouville space propagator to quantum dissipative dynamics. *J. Chem. Phys.* 130:084105. https://doi.org/10.1063/1.3077918.

52. van der Vorst, H. A. 1992. Bi-CGSTAB: A Fast and Smoothly Converging Variant of Bi-CG for the Solution of Nonsymmetric Linear Systems. *SIAM J. Sci. Comput.* 13:631–644. https://doi.org/10.1137/0913035.

53. Mukai, K., S. Abe, and H. Sumi. 1999. Theory of rapid excitation-energy transfer from B800 to optically-forbidden exciton states of B850

in the antenna system LH2 of photosynthetic purple bacteria. *J. Phys. Chem. B.* 103:6096–6102. https://doi.org/10.1021/jp984469g.

54. Scholes, G. D., and G. R. Fleming. 2000. On the mechanism of light harvesting in photosynthetic purple bacteria: B800 to B850 energy transfer. *J. Phys. Chem. B.* 104:1854–1868. https://doi.org/10.1021/jp993435l.
55. Renger, T. 2009. Theory of excitation energy transfer: from structure to function. *Photosynth. Res.* 102:471–485. https://doi.org/10.1007/s11120-009-9472-9.
56. Renger, T., and R. A. Marcus. 2002. On the relation of protein dynamics and exciton relaxation in pigment–protein complexes: An estimation of the spectral density and a theory for the calculation of optical spectra. *J. Chem. Phys.* 116:9997–10019. https://doi.org/10.1063/1.1470200.
57. Yang, M., and G. R. Fleming. 2002. Influence of phonons on exciton transfer dynamics: comparison of the Redfield, Förster, and modified Redfield equations. *Chem. Phys.* 275:355–372. https://doi.org/10.1016/S0301-0104(01)00540-7.
58. Chachisvilis, M., O. Kühn, …, V. Sundström. 1997. Excitons in photosynthetic purple bacteria: wavelike motion or incoherent hopping? *J. Phys. Chem. B.* 101:7275–7283. https://doi.org/10.1021/jp963360a.
59. Fassioli, F., R. Dinshaw, …, G. D. Scholes. 2014. Photosynthetic light harvesting: excitons and coherence. *J. R. Soc. Interface.* 11:20130901.
60. Cantor, R. S. 1997. Lateral pressures in cell membranes: a mechanism for modulation of protein function. *J. Phys. Chem. B.* 101:1723–1725. https://doi.org/10.1021/jp963911x.
61. Ding, W., M. Palaiokostas, …, M. Orsi. 2015. Effects of lipid composition on bilayer membranes quantified by all-atom molecular dynamics. *J. Phys. Chem. B.* 119:15263–15274. https://doi.org/10.1021/acs.jpcb.5b06604.
62. Garland, T. 2014. Trade-offs. *Curr. Biol.* 24:R60–R61. https://doi.org/10.1016/j.cub.2013.11.036.
63. Johansson, M., J. Zhang, and M. Ehrenberg. 2012. Genetic code translation displays a linear trade-off between efficiency and accuracy of tRNA selection. *Proc. Natl. Acad. Sci. USA.* 109:131–136. https://doi.org/10.1073/pnas.1116480109.
64. Andersen, O. S., and R. E. Koeppe. 2007. Bilayer thickness and membrane protein function: an energetic perspective. *Annu. Rev. Biophys. Biomol. Struct.* 36:107–130. https://doi.org/10.1146/annurev.biophys.36.040306.132643.
65. Zhang, H.-D., and Y. Yan. 2016. Kinetic rate kernels via hierarchical Liouville–space projection operator approach. *J. Phys. Chem. A.* 120:3241–3245.
66. Harel, E., and G. S. Engel. 2012. Quantum coherence spectroscopy reveals complex dynamics in bacterial light-harvesting complex 2 (LH2). *Proc. Natl. Acad. Sci. USA.* 109:706–711. https://doi.org/10.1073/pnas.1110312109.
67. Cao, J., and R. J. Silbey. 2009. Optimization of exciton trapping in energy transfer processes. *J. Phys. Chem. A.* 113:13825–13838. https://doi.org/10.1021/jp9032589.
68. Wu, H.-M., S. Savikhin, …, G. J. Small. 1996. Femtosecond and hole-burning studies of B800's excitation energy relaxation dynamics in the LH2 antenna complex of Rhodopseudomonas acidophila (strain 10050). *J. Phys. Chem.* 100:12022–12033. https://doi.org/10.1021/jp9608178.
69. De Caro, C., R. W. Visschers, …, S. Völker. 1994. Inter-and intraband energy transfer in LH2-antenna complexes of purple bacteria. A fluorescence line-narrowing and hole-burning study. *J. Phys. Chem.* 98:10584–10590. https://doi.org/10.1021/j100092a032.
70. Monshouwer, R., I. O. de Zarate, …, R. van Grondelle. 1995. Low-intensity pump-probe spectroscopy on the B800 to B850 transfer in the light harvesting 2 complex of Rhodobacter sphaeroides. *Chem. Phys. Lett.* 246:341–346. https://doi.org/10.1016/0009-2614(95)01104-H.
71. Creatore, C., M. A. Parker, …, A. W. Chin. 2013. Efficient biologically inspired photocell enhanced by delocalized quantum states. *Phys. Rev. Lett.* 111:253601. https://doi.org/10.1103/PhysRevLett.111.253601.
72. Higgins, K. D. B., B. W. Lovett, and E. M. Gauger. 2017. Quantum-enhanced capture of photons using optical ratchet states. *J. Phys. Chem. C.* 121:20714–20719. https://doi.org/10.1021/acs.jpcc.7b07138.
73. Zhang, Y., A. Wirthwein, …, S. Kais. 2016. Dark states enhance the photocell power via phononic dissipation. *Phys. Chem. Chem. Phys.* 18:31845–31849. https://doi.org/10.1039/C6CP06098F.

Biophysical Journal, Volume *124*

Supplemental information

Theory of photosynthetic membrane influence on B800-B850 energy transfer in the LH2 complex

Chawntell Kulkarni, Hallmann Óskar Gestsson, Lorenzo Cupellini, Benedetta Mennucci, and Alexandra Olaya-Castro

Supporting material

Theory of photosynthetic membrane influence on B800-B850 energy transfer in the LH2 complex

Chawntell Kulkarni[1], Hallmann Óskar Gestsson[2], Lorenzo Cupellini[3], Benedetta Mennucci[4], and Alexandra Olaya-Castro[5]

[1, 2, 5]Department of Physics and Astronomy, University College London, London WC1E 6BT, United Kingdom

[3, 4]Dipartimento di Chimica e Chimica Industriale, Università di Pisa, Via G. Moruzzi 13, 56124 Pisa, Italy

Modified Redfield theory

The energy transfer rates computed with generalised Förster theory (GFT) use lineshape functions that are derived perturbatively as introduced in the generalised Förster theory section. The exciton lifetime τ_α that enters the lineshape functions is given by

$$\tau_\alpha = \left(\frac{1}{2} \sum_{\alpha \neq \beta} k_{\alpha\beta}^{\mathrm{MR}} \right)^{-1}, \tag{1}$$

where $k_{\alpha\beta}^{\mathrm{MR}}$ is the energy transfer rate from a donor exciton α to all possible acceptor excitons β, given that they are localised on the same ring as the donor exciton. Since the interchromophore electronic couplings within each ring are strong, modified Redfield theory is used to obtain the intra-ring exciton transfer rates. By assuming that the electronic states of each ring are weakly coupled to their environment, H_{SB} is treated as a perturbation on the dynamics within each ring.

The modified Redfield energy transfer rate between two excitons in the same ring is given by [1]

$$\begin{aligned} k_{\alpha\beta}^{\mathrm{MR}} = 2\mathrm{Re} \int_0^\infty dt \; & e^{-i\omega_{\alpha\beta} t} e^{-i(\lambda_{\alpha\alpha,\alpha\alpha} + \lambda_{\beta\beta,\beta\beta})t} e^{-g_\alpha(t) - g_\beta(t)} e^{2g_{\beta\beta,\alpha\alpha} + 2i\lambda_{\beta\beta,\alpha\alpha}} \\ & \times \left[\ddot{g}_{\beta\alpha,\beta\alpha}(t) - (\dot{g}_{\beta\alpha,\beta\beta}(t) - \dot{g}_{\beta\alpha,\alpha\alpha}(t) + 2i\lambda_{\beta\alpha,\beta\beta})^2 \right], \end{aligned} \tag{2}$$

where the terms have been defined in the main text (generalised Förster theory section).

Propagation of the dipole operator

Numerical computation of the absorption and fluorescence expressions given in the Linear spectra section using HEOM theory is achieved by rewriting the auto-correlation as

$$\langle \hat{\mu}_p(t)\hat{\mu}_p(t)\rangle_\rho = \mathrm{Tr}(\hat{\mu}_p e^{\mathcal{L}t}[\hat{\mu}_p\hat{\rho}]), \tag{3}$$

where $\mathcal{L}$ is the HEOM generator of dynamics and $\hat{\rho}$ is the reduced system density matrix. The half-sided Fourier transform is then formally calculated to give

$$\int_0^\infty \mathrm{d}t \hat{\mu}_p e^{\mathcal{L}t}[\hat{\mu}_p\hat{\rho}]e^{i\omega t} = -\hat{\mu}_p \frac{1}{\mathcal{L}+i\omega}[\hat{\mu}_p\hat{\rho}]. \tag{4}$$

We numerically determine $\hat{x}_{p,\omega} = \frac{1}{\mathcal{L}+i\omega}[\hat{\mu}_p\hat{\rho}]$ by solving the linear system $(\mathcal{L}+i\omega)[\hat{x}_{p,\omega}] = \hat{\mu}_p\hat{\rho}$ using the BiCGSTAB Krylov subspace method [2]. This method of numerically computing spectra is more efficient than numerically Fourier transforming the dynamics as a result of the sparsity of the matrix representation for $\mathcal{L}$.

Finding the thermal state

The fluorescence spectra in the Linear spectra section is computed by performing a trace with respect to the thermal state $\hat{\rho}_{th}$, which satisfies the property $\mathcal{L}\hat{\rho}_{th} = 0$. In order to determine the thermal state we solve this linear system using the BiCGSTAB method [2] which is supplied with an initial guess given by the Boltzmann state $e^{-\beta\hat{H}}/\mathrm{Tr}(e^{-\beta\hat{H}})$. Doing so guarantees that the solver will not yield the trivial zero matrix solution, which of course does not represent a physical state.

Donor acceptor exciton energy gap

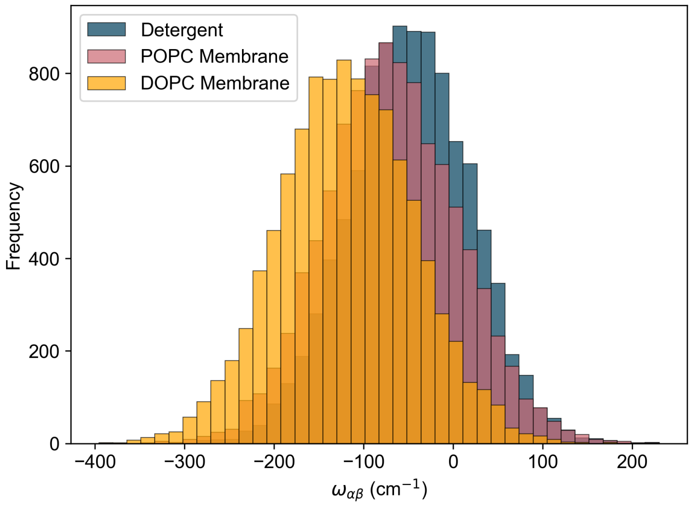

Figure S1: Distribution of 10,000 realisations of the energy gap between the B800 and B850 exciton forming the dominant exciton energy transfer pathway in detergent isolated LH2, LH2 embedded in a DOPC membrane and in a POPC membrane. Despite different levels of static disorder in membrane and detergent environments, the distribution of the energy gap of the dominant pathway in B800 to B850 transfer remains similar in each environment.

B800 to B850 transfer rate distributions

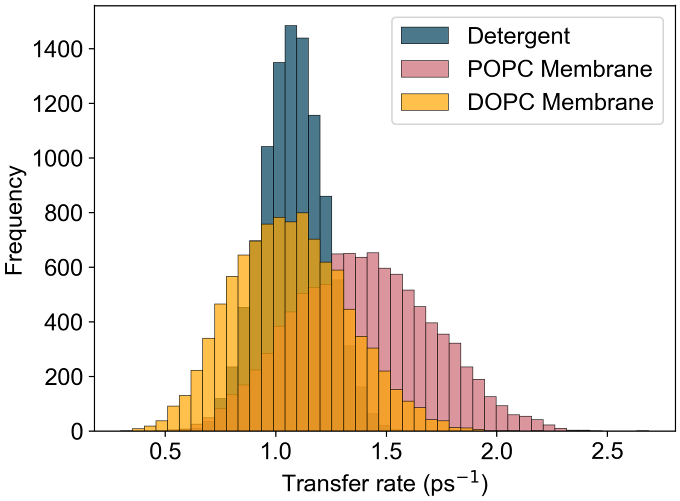

Figure S2: Distribution of 10,000 realisations of the B800 to B850 energy transfer rate calculated using GFT for detergent isolated LH2, and two different lipid compositions of membrane embedded LH2, DOPC and POPC. The same static disorder parameters were used to calculate rates in all three environments. Average transfer rates are 1.08 ps^{-1}, 1.07 ps^{-1} and 1.40 ps^{-1} respectively.

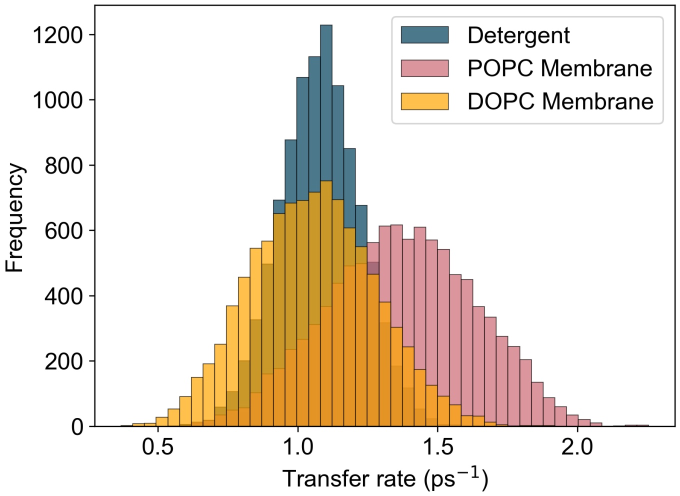

Figure S3: (a) Distribution of 10,000 realisations of the B800 to B850 energy transfer rate calculated using GFT for detergent isolated LH2, and two different lipid compositions of membrane embedded LH2, DOPC and POPC. The same spectral density was used for all three environments. Average transfer rates are 1.08 ps^{-1}, 1.05 ps^{-1} and 1.37 ps^{-1} respectively.

Linear spectra for alternative Hamiltonian parameters

To select Hamiltonian parameters that describe LH2 in detergent, we conducted an extensive review of the different parameter sets reported in the literature. For membrane LH2, to our knowledge, no reliable parameter sets other than the ones reported by some of us [3, 4] are available. Parameters for detergent-isolated LH2 computed using the same quantum chemical methods as those used for membrane-embedded LH2 are currently not available, and their computation requires additional developments outside the scope of this study. Existing parameter sets are either derived from first principles or from experimental spectroscopic measurements [5]. To assess which parameter set is suitable to describe detergent-solubilised LH2, we compare the linear spectra predicted by each set to what is measured in experimental. Specifically, we compare the predicted redshift of the B850 absorption peak in membrane relative to the peak position in detergent, as this is the key observation in experimental work comparing LH2 solubilised in detergent to LH2 in a lipid environment [6, 7, 8].

We have selected parameter sets for LH2 from the literature that have each been derived using different standard methodologies and compute linear absorption and fluorescence spectra using the hierarchical equations of motion (HEOM). We compare parameters derived using point dipole approximation (PDA) [9], point monopole approximation (PMA) [10] and transition density cubes method (TDCM)[11] to the parameters used in our study which have been derived from experimental spectra of the LH2 [12] The PDA and TDCM derived parameters do not have site energy values hence we use the site energies from the detergent model used in the paper. The most important aspect are the electronic couplings which we have shown underlie the observed changes in energy transfer rates and spectra from detergent to membrane.

Figure S4(a) shows the experimental spectra extracted from Figure 1(b) in the study by Ogren *et al.* [6] and Figures S4(b-d) show the spectra computed using each of the detergent parameter sets (shown in green) along with the spectra computed with the detergent and membrane parameters used in our study (shown in blue and red, respectively).

All three parameter sets predict the redshift of the B850 absorption peak in membrane compared to detergent despite each being derived using different methodologies. However, the predicted size of the redshift varies from one model to another which may result in changes in the B800 to B850 transfer rates that each model predict. We find that the size of the redshift predicted by the detergent model that we use in our study best compares with the experimentally observed shift.

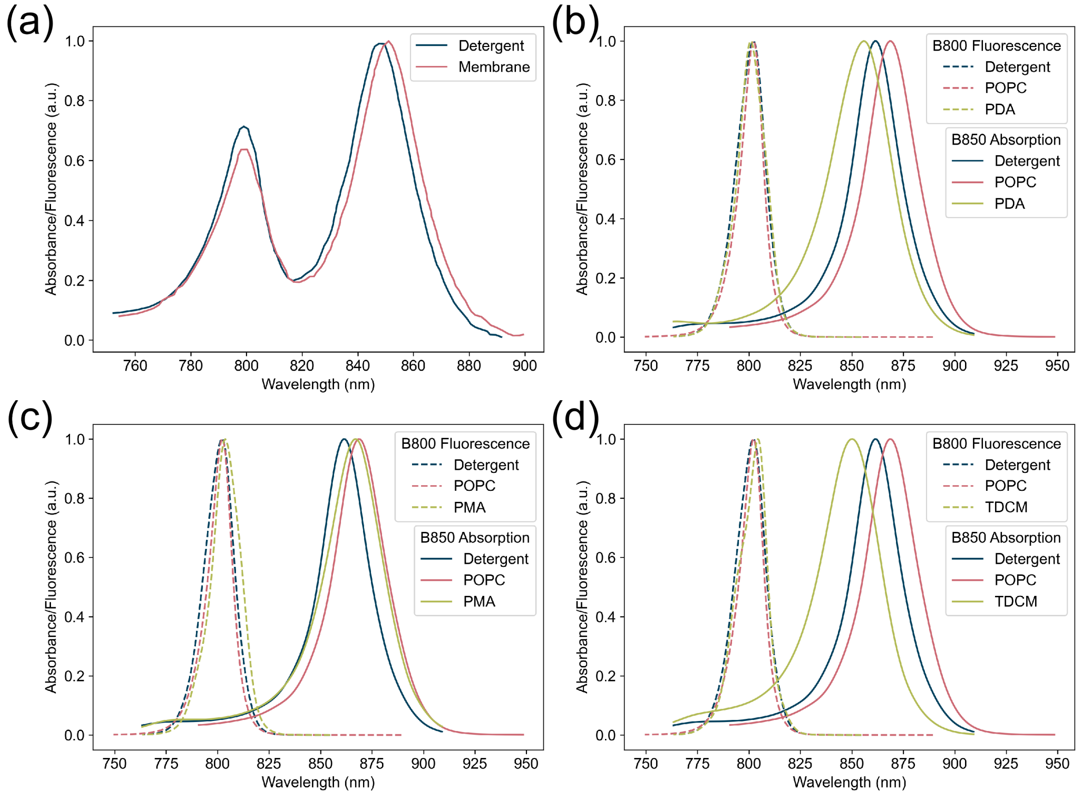

Figure S4: (a) Experimental absorption spectra of the LH2 from *Rhodobacter sphaeroides* in detergent and in membrane extracted from Figure 1(b) in Ogren *et al.* [6]. (b-d) Absorption spectra of the B850 ring and fluorescence spectra of the B800 ring for the Detergent and POPC membrane models used in the paper calculated using HEOM with truncation 3 for absorption and truncation 4 for fluorescence. The spectra are averaged over 1000 realisations of static disorder. For comparison a second detergent model is given in each plot where the electronic parameters have been computed using (b) point dipole approximation [9], (c) point monopole approximation [10] (d) and transition density cubes method [11].

B800 to B850 energy transfer rate for alternative Hamiltonian parameters

In the previous section, we showed how different Hamiltonian parameters predict varying degrees of redshift of the B850 peak from detergent to membrane, the redshift being the key observation in experimental spectroscopic measurements. The varying position of the B850 band predicted by each detergent parameter set, mean changes in the overlap of the B800 fluorescence and B850 absorption, which may result in differences in the B800 to B850 transfer rate predicted by each model.

Experimental pump-probe measurements find a 30% increase in B800 to B850 transfer rate from LH2 in detergent to LH2 in a lipid environment [6]. Using GFT to compute the B800 to B850 transfer rate averaged over 10,000 realisations, we compare the change in rate from detergent to membrane predicted by each of the detergent parameter sets, to what is measured in experiment.

Table S1 lists the B800 to B850 transfer rate predicted by each of the detergent parameter sets and the percentage change in transfer rate from detergent to membrane POPC. The alternative parameter sets show significant qualitative differences compared to experimental findings. The PMA and PDA parameters predict that the average transfer rate nearly doubles from detergent to membrane, while the TDCM parameters predict that energy transfer is actually slower in membrane. In contrast, the detergent parameters chosen for our study predict a 24% increase in the transfer rate, in qualitative agreement with experimental observations. Thus, the chosen set of parameters for detergent qualitatively capture the fundamental spectral and dynamical changes in the LH2 from detergent to lipid membrane environments observed in experiment, making the selected model an ideal basis to advance our understanding of the microscopic changes occurring within the complex in it's native physiological environment.

Parameter set	Membrane POPC	Detergent	PMA	PDA	TDCM
Transfer rate (ps^{-1})	1.34	1.08	0.69	0.67	1.52
% increase in rate in membrane	n/a	24%	94%	100%	-11%

Table S1: Average B800 to B850 transfer rates computed using GFT with 10,000 realisations of disorder for various Hamiltonian parameters. The parameter sets are, as listed from left to right, the membrane POPC and detergent parameters used in our study, alternative detergent parameters computed using point monopole approximation, point dipole approximation and transition density cubes method. The last row gives the percentage change in transfer rate from each of the alternative detergent models to membrane POPC.

Standard deviation of exciton energies

Exciton	Membrane	Detergent
k = 9	111	93
k = +8	87	73
k = -8	80	67
k = +7	76	64
k = -7	75	62
k = +6	74	62
k = -6	73	61
k = +5	73	62
k = -5	72	64
k = +4	71	60
k = -4	72	59
k = +3	71	59
k = -3	74	60
k = +2	72	58
k = -2	75	60
k = +1	74	59
k = -1	79	63
k = 0	91	65

Table S2: Standard deviations of the B850 exciton energy levels in cm^{-1} computed using 10,000 realisations of disorder listed from highest to lowest energy.

Exciton	Membrane	Detergent
D = 8	19	28
D = 7	16	22
D = 6	15	20
D = 5	15	19
D = 4	15	19
D = 3	15	19
D = 2	15	20
D = 1	16	21
D = 0	19	26

Table S3: Standard deviations of the B800 exciton energy levels in cm^{-1} computed using 10,000 realisations of disorder listed from highest to lowest energy.

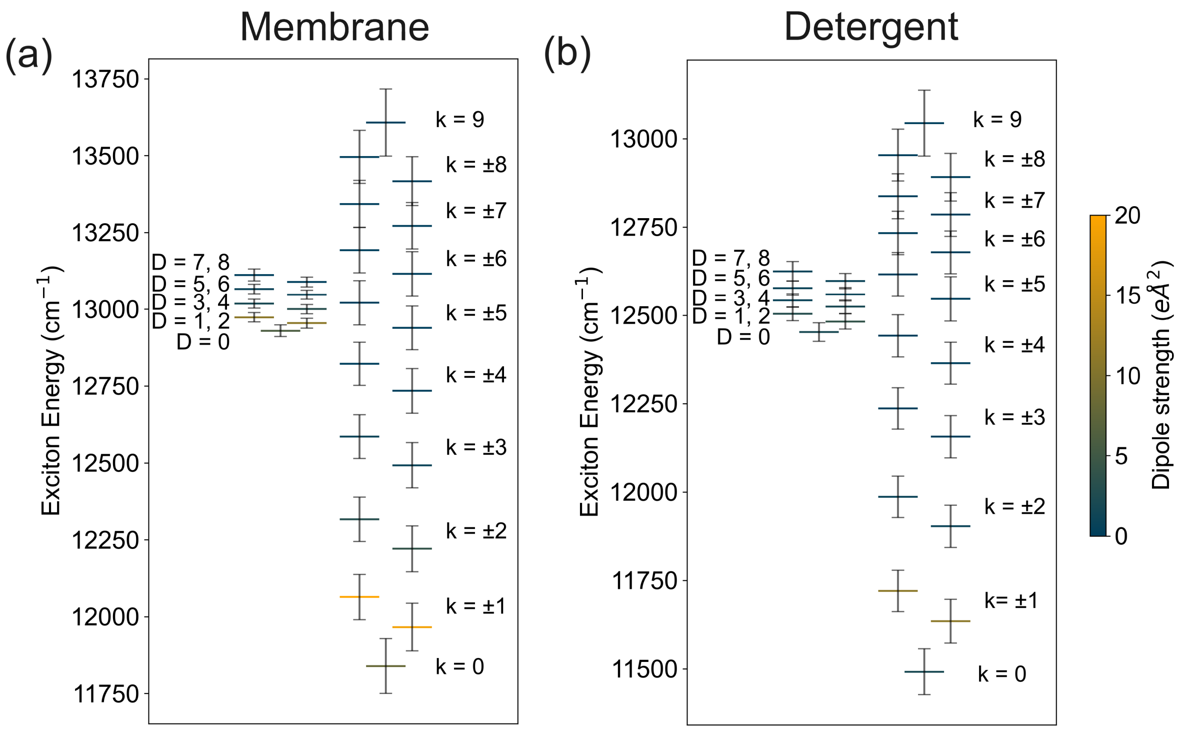

Figure S5: Average positions of the exciton energy levels of the B800 and B850 rings of (a) membrane embedded LH2 and (b) detergent isolated LH2 averaged over 10,000 realisations of static disorder. The disorder expected in each ring for each environment is given in Table S2. Standard deviations for each level are given as error bars.

Supporting References

[1] Mino Yang and Graham R Fleming. Influence of phonons on exciton transfer dynamics: comparison of the redfield, förster, and modified redfield equations. *Chem. Phys.*, 282(1):163–180, 2002.

[2] H. A. van der Vorst. Bi-cgstab: A fast and smoothly converging variant of bi-cg for the solution of nonsymmetric linear systems. *SIAM J. Sci. Comput.*, 13(2):631–644, 1992.

[3] Lorenzo Cupellini, Sandro Jurinovich, Marco Campetella, Stefano Caprasecca, Ciro A Guido, Sharon M Kelly, Alastair T Gardiner, Richard Cogdell, and Benedetta Mennucci. An ab initio description of the excitonic properties of lh2 and their temperature dependence. *J. Phys. Chem. B.*, 120(44):11348–11359, 2016.

[4] Felipe Cardoso Ramos, Michele Nottoli, Lorenzo Cupellini, and Benedetta Mennucci. The molecular mechanisms of light adaption in light-harvesting complexes of purple bacteria revealed by a multiscale modeling. *Chem. Sci.*, 10(42):9650–9662, 2019.

[5] Sergei Tretiak, Chris Middleton, Vladimir Chernyak, and Shaul Mukamel. Bacteriochlorophyll and carotenoid excitonic couplings in the lh2 system of purple bacteria. *J. Phys. Chem. B.*, 104(40):9540–9553, 2000.

[6] John I Ogren, Ashley L Tong, Samuel C Gordon, Aurélia Chenu, Yue Lu, Robert E Blankenship, Jianshu Cao, and Gabriela S Schlau-Cohen. Impact of the lipid bilayer on energy transfer kinetics in the photosynthetic protein lh2. *Chem. Sci.*, 9(12):3095–3104, 2018.

[7] Arvi Freiberg, Margus Rätsep, and Kõu Timpmann. A comparative spectroscopic and kinetic study of photoexcitations in detergent-isolated and membrane-embedded lh2 light-harvesting complexes. *Biochimica et Biophysica Acta (BBA)-Bioenergetics*, 1817(8):1471–1482, 2012.

[8] Ritesh Agarwal, Abbas H Rizvi, Bradley S Prall, John D Olsen, C Neil Hunter, and Graham R Fleming. Nature of disorder and inter-complex energy transfer in lh2 at room temperature: a three pulse photon echo peak shift study. *J. Phys. Chem. A.*, 106(33):7573–7578, 2002.

[9] Villy Sundström, Tõnu Pullerits, and Rienk van Grondelle. Photosynthetic light-harvesting: reconciling dynamics and structure of purple bacterial lh2 reveals function of photosynthetic unit, 1999.

[10] Kenneth Sauer, Richard J Cogdell, Steve M Prince, Andy Freer, Neil W Isaacs, and Hugo Scheer. Structure-based calculations of the optical spectra of the lh2 bacteriochlorophyll-protein complex from rhodopseudomonas acidophila. *Photochem. Photobiol.*, 64(3):564–576, 1996.

[11] Brent P Krueger, Gregory D Scholes, and Graham R Fleming. Calculation of couplings and energy-transfer pathways between the pigments of lh2 by the ab initio transition density cube method. *J. Phys. Chem. B.*, 102(27):5378–5386, 1998.

[12] Arvi Freiberg, Margus Rätsep, Kõu Timpmann, and Gediminas Trinkunas. Excitonic polarons in quasi-one-dimensional lh1 and lh2 bacteriochlorophyll a antenna aggregates from photosynthetic bacteria: A wavelength-dependent selective spectroscopy study. *Chem. Phys.*, 357(1-3):102–112, 2009.
